# Engineered Extracellular Vesicle‐Delivered CRISPR/CasRx as a Novel RNA Editing Tool

**DOI:** 10.1002/advs.202206517

**Published:** 2023-02-02

**Authors:** Tianwen Li, Liansheng Zhang, Tao Lu, Tongming Zhu, Canbin Feng, Ni Gao, Fei Liu, Jingyu Yu, Kezhu Chen, Junjie Zhong, Qisheng Tang, Quan Zhang, Xiangyang Deng, Junwei Ren, Jun Zeng, Haibo Zhou, Jianhong Zhu

**Affiliations:** ^1^ Department of Neurosurgery Huashan Hospital Shanghai Medical College Fudan University National Center for Neurological Disorders National Key Laboratory for Medical Neurobiology Institutes of Brain Science Shanghai Key Laboratory of Brain Function and Regeneration Institute of Neurosurgery MOE Frontiers Center for Brain Science Shanghai 200040 China; ^2^ Institute of Neuroscience State Key Laboratory of Neuroscience Key Laboratory of Primate Neurobiology CAS Center for Excellence in Brain Science and Intelligence Technology Shanghai Research Center for Brain Science and Brain‐Inspired Intelligence Shanghai Institutes for Biological Sciences Chinese Academy of Sciences Shanghai 200031 China; ^3^ Anhui Province Key Laboratory of Clinical and Preclinical Research in Respiratory Disease Molecular Diagnosis Center Department of Pulmonary and Critical Care Medicine First Affiliated Hospital Bengbu Medical College No. 287 Changhuai Road Bengbu Anhui 233004 China

**Keywords:** CRISPR/CasRx, extracellular vesicles, inflammatory disease, RNA editing

## Abstract

Engineered extracellular vesicles (EVs) are considered excellent delivery vehicles for a variety of therapeutic agents, including nucleic acids, proteins, drugs, and nanomaterials. Recently, several studies have indicated that clustered regularly interspaced short palindromic repeats (CRISPR)/CRISPR‐associated 9 (Cas9) delivered by EVs enable efficient DNA editing. However, an RNA editing tool delivered by EVs is still unavailable. Here, a signal peptide‐optimized and EVs‐delivered guide RNA (gRNA) and CRISPR/CasRx (Cas13d) system capable of rapidly inhibiting the expression of targeted genes with quick catabolism after performing their functions is developed. EVs with CRISPR/CasRx and tandem gRNAs targeting pivotal cytokines are further packed whose levels increase substantially over the course of acute inflammatory diseases and find that these engineered EVs inhibit macrophage activation in vitro. More importantly, this system attenuates lipopolysaccharide (LPS)‐triggered acute lung injury and sepsis in the acute phase, mitigating organ damage and improving the prognosis in vivo. In summary, a potent tool is provided for short‐acting RNA editing, which could be a powerful therapeutic platform for the treatment of acute diseases.

## Graphical abstract

In this study, a signal peptide‐optimized and extracellular vesicles‐delivered CRISPR/CasRx and guide RNA system is developed, capable of rapidly inhibiting the expression of multiple inflammatory cytokines with quick catabolism after performing their functions both in vitro and in vivo, providing a short‐acting RNA editing tool for the treatment of acute inflammatory diseases.

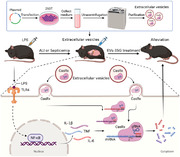

## 1 Introduction

Clustered regularly interspaced short palindromic repeats (CRISPR)/CRISPR‐associated (CRISPR/Cas) systems originating from the adaptive immune system of bacteria and archaea are powerful tools to efficiently manipulate DNA and RNA.^[^ ^1^ ^]^ A major obstacle for the widespread translational application of the CRISPR/Cas system is the lack of a proper and effective delivery method. Although viral vectors are the dominant method of delivery,^[^ ^2^ ^]^ the immunogenicity and hysteresis (the targeted cells must transcribe and translate the CRISPR/Cas sequences into proteins first) of these viral vectors greatly limits the translational application of the CRISPR/Cas system.^[^ ^2b^ ^]^

Extracellular vesicles (EVs) were initially presumed to act as a vehicle to mediate cell‐to‐cell communication locally and between organs in physiological or pathological processes.^[^ ^3^ ^]^ EVs are closely related to the biological regulation, synthesis, and metabolism of diverse systems and organs, and they also play essential roles in the progression of various diseases.^[^ ^3^ ^]^ Various cargos from the original cell, including RNA, DNA, proteins, and metabolites, can be enveloped and delivered by EVs and then transported to neighboring or distant cells. With the development of biotechnology and research methods, natural EVs can no longer meet our therapeutic needs. Although many unsolved mysteries in terms of EV synthesis and function persist that require further exploration, many engineered and customized EVs have been developed and applied for therapeutic purposes.^[^ ^4^ ^]^ Recent studies of engineered EVs have shown that the genome‐editing CRISPR‐Cas9/guide RNA (gRNA) complex delivered by EVs performs gene editing efficiently after entering target cells.^[^ ^5^ ^]^ Compared with the CRISPR/Cas9 system, which causes permanent genetic alterations, CRISPR/Cas13 is a relatively safer tool because it directly recognizes and cleaves target RNA instead of genomic DNA and can be applied to transiently affect the transcriptome.^[^ ^6^ ^]^ CRISPR/Cas13d (CasRx) has high specificity and efficiency compared with the RNAi system, which supports its further development and widespread application.^[^ ^6^, ^7^ ^]^ However, no reports of the use of EVs to deliver CasRx to prevent or treat disease have been published.

In the present study, we optimized the EV‐mediated protein delivery system to achieve an easier and more efficient method for CRISPR/CasRx loading. Notably, the CasRx‐gRNA complex delivered by EVs successfully disrupted the RNA of both exogenous and endogenous genes in a short‐acting manner and ultimately reduced the expression of target proteins. This versatile tool is particularly useful for transient interventions in acute diseases. We used a dual plasmid cotransfection system to achieve an effective gRNA screen and further revealed that simultaneous knockdown of three pivotal cytokines (interleukin‐6 (IL‐6), tumor necrosis factor (TNF) and interleukin‐1*β* (IL‐1*β*)) by engineered EVs reduced macrophage activation. In animal models of acute lung injury (ALI) and sepsis, these engineered EVs were tracked using in vivo imaging and immunohistochemistry and were proven to reduce mortality and mitigate organ damage in the acute phase. Thus, the EVs‐delivered CasRx/gRNA complex rapidly mediates the efficient inhibition of targeted genes and provides a platform for the perturbation of certain gene(s), and the treatment of acute diseases or acute stages of certain serious diseases.

## 2 Results

### 2.1 Optimized Packaging of CasRx in EVs with the tPA Ligand

**Figure 1:**
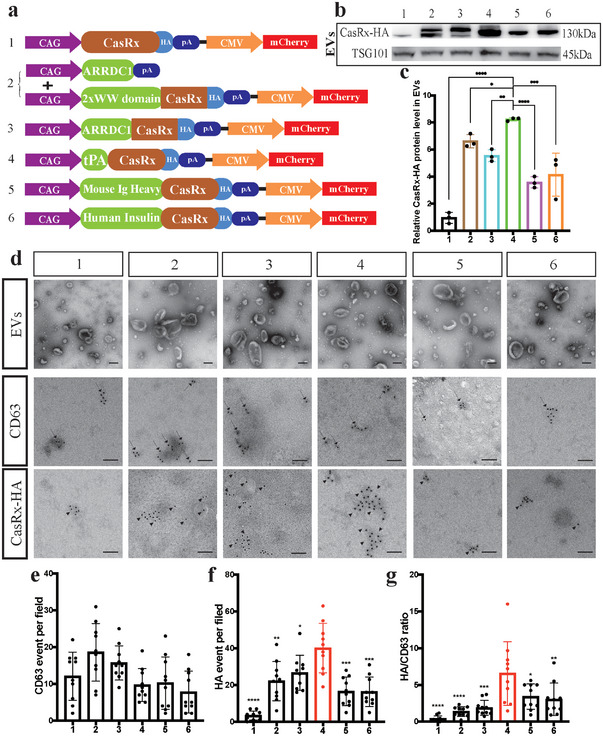
The tPA ligand improves protein loading into EVs. a) A schematic of the CasRx vector with different loading ligands. CasRx was labeled with an HA‐tag. b) Immunoblotting analysis comparing CasRx‐HA levels in EVs from different groups, TSG101 served as a control. c) Statistical analysis of the relative CasRx‐HA protein level in EVs. d) Morphological specificity of EVs examined using TEM (upper panel). Representative images of CD63 (middle panel) and CasRx‐HA (lower panel) staining in EVs from different groups obtained using IEM. Scale bar = 100 nm (upper panel) or 50 nm (middle and lower panels). e)Statistical analysis of CD63 counts per field (*n* = 10 wells per group). f) Statistical analysis of CasRx‐HA counts per field (*n* = 10 wells per group). g) Statistical analysis of the CasRx‐HA/CD63 ratio per field (*n* = 10 wells per group) showing that the tPA ligand group had the highest ratio of CasRx‐HA to CD63. ****p < 0.0001, ***p < 0.001, **p < 0.01, and *p < 0.05. The data are presented as the means ± SD.

Recent studies have shown that the loading of target proteins into EVs is significantly improved by ligand pairing.^[^ ^5^, ^8^ ^]^ The reported methods were similar and verified that the target proteins can be transported extracellularly through EVs when coupled with the key proteins involved in EV synthesis. We questioned whether the short signal peptides needed for protein secretion, which direct the target proteins to the extracellular space, would achieve the same purpose. We selected tPA,^[^ ^9^ ^]^ mouse Ig heavy chain,^[^ ^10^ ^]^ and human insulin^[^ ^11^ ^]^ as these three typical signal peptides to construct the CasRx plasmids and compared their functions using the widely used arrestin domain‐containing protein 1 (ARRDC1)‐mediated microvesicle (ARMM) platform,^[^ ^5d^ ^]^ which relies on 2 trans complementing constructions of the ARRDC1 and WW domains. CasRx in the control group had no ligand (group #1); in other groups, all ligands were fused to CasRx at the N‐terminus. In addition, the HA‐tag was fused to the C‐terminus of CasRx for tracking (**Figure** **1**a). The engineered EVs were extracted from HEK293T cells using an established method (Figure S1, Supporting Information). Notably, the procedures used for extracting and purifying EVs are different from those for regular proteins.^[^ ^5^, ^9^, ^10^, ^12^ ^]^ Certain tagged proteins, various salt‐containing agents or chromatography separation columns are necessary to extract and purify the proteins. However, the extraction of EVs requires an ultracentrifugation process, in which most dissociative proteins (outside of EVs) are discarded (Figure S1, Supporting Information). Therefore, the tPA‐CasRx (group #4), mouse Ig heavy chain‐CasRx (group #5), and the human insulin‐CasRx (group #6) proteins were mainly derived from EVs. EVs were collected separately, and the immunoblotting results showed that regardless of the ligand, the amount of CasRx protein in EVs was substantially increased compared with that in the control group, and the tPA group contained the largest amount of packaged CasRx (Figure 1b,c). In addition, signal peptides (groups #4, #5, and #6) significantly increased CasRx expression in the cell lysate compared with the control (group #1) and ARRDC1 platforms (group #2) (Figure S2a,b, Supporting Information).

We conducted nanoparticle tracking analysis (NTA) of these EVs from different groups to compare EV secretion to further characterize the engineered EVs. Interestingly, naïve EVs only presented one peak in the NTA image (Figure S2c, Supporting Information), while all engineered EVs presented double peaks (Figure S2d‐i, Supporting Information), which implied the loading of CasRx. In addition, the mean particle size of all six groups that transfected with plasmids was larger than that of naïve EVs (Figure S2c–j, Supporting Information, #1‐#6≥130 nm, naïve EVs < 125 nm). The zeta potential of each group was ‐20.30 ± 0.65 mV (naïve EVs), ‐15.43 ± 2.02 mV (#1), ‐16.01 ± 0.91 mV (#2), ‐12.50 ± 0.94 mV (#3), ‐15.72 ± 0.53 mV (#4), ‐21.93 ± 0.61 mV (#5), and ‐17.33 ± 0.24 mV (#6) (Figure S2c–j, Supporting Information). We also measured their polydispersity index (PDI), which ranged from 0.47 to 0.82 (Figure S2j, Supporting Information).

Moreover, the transmission electron microscopy (TEM) results showed that the engineered EVs from these six groups exhibited no remarkable morphological differences (Figure 1d, lane 1). In addition, the background of all groups was very clear, and no other dissociative proteins were detected, indicating that CasRx was enveloped in EVs (Figure 1d, lane 1). We performed an immunogold electron microscopy (IEM) assay to further examine the CasRx load, and the tPA group had the largest CasRx load (Figure 1d,f). Using the CasRx/CD63 ratio to normalize the CasRx load in EVs, we verified that tPA was the best ligand for CasRx (Figure 1e,g) in our system. In addition, the IEM results were largely consistent with the immunoblotting results (Figure 1b,c). Thus, these results showed that the signal peptide at the N‐terminus packaged target proteins into EVs and suggested that the tPA ligand enveloped more target proteins into EVs.

**Figure 2:**
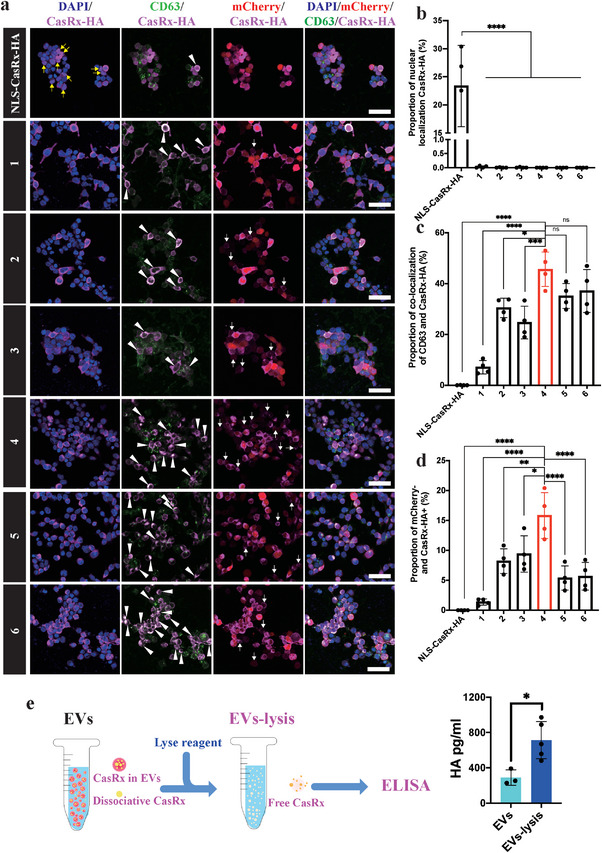
EVs‐mediated CasRx delivery in cells. a) Immunostaining with DAPI, CD63, CasRx‐HA, and mCherry in HEK293T cells transfected with the target plasmids. Representative images showed the nuclear localization of CasRx (yellow arrows), colocalization of CD63 and CasRx (white arrowheads), and mCherry‐ CasRx+ cells (white arrows). Scale bar = 50 µm. b) Statistical analysis of the proportion of cells with nuclear localization of CasRx, *n* = 4 wells per group. c) Statistical analysis of the proportion of cells with colocalization of CD63 and CasRx, *n* = 4 wells per group. d) Statistical analysis of the proportion of mCherry‐CasRx+ cells, *n* = 4 wells per group. e) Schematic image (left) and statistical results (right) of CasRx‐HA ELISA. ****p < 0.0001, ***p < 0.001, **p < 0.01, and *p < 0.05. The data are presented as the means ± SD.

We conducted immunocytochemical (ICC) staining of HEK293T cells transfected with these different plasmids to further verify that CasRx was surely loaded in EVs. The nuclear localization signal (NLS) is the most commonly used signal peptide in gene editing, and we included it as a control in the comparison. As expected, CasRx exhibited no nuclear colocalization, except in the NLS group (**Figure** **2**a,b). Furthermore, CasRx colocalized with the EV marker CD63 in groups #1 to #6, but little colocalization was observed in the NLS group. Among the groups, the tPA group exhibited the strongest colocalization of CasRx and CD63 (Figure 2a,c). Notably, once the HEK293T cells were transfected with the plasmids, they were expected to express both CasRx and mCherry, indicating that the CasRx proteins of CasRx^+^ mCherry^−^ cells must originate from transfected cells. In other words, these CasRx proteins were not produced by the cell itself but were transferred from other transfected cells. Therefore, the CasRx^+^ mCherry^−^ ratio also indicated the packaging capacity of different ligands to a certain extent. Similar to the IEM and immunoblotting results, the tPA group had the highest CasRx^+^ mCherry^−^ ratio (Figure 2a,d). Then, we detected the amount of dissociated CasRx in the tPA group using an enzyme‐linked immunosorbent assay (ELISA), which showed that more than two‐thirds of CasRx was enveloped in EVs (Figure 2e). Coomassie Brilliant Blue staining showed that the CasRx proteins accounted for 12% of all proteins (Figure S3a,b, Supporting Information).

Taken together, our results showed that CasRx is efficiently packaged into EVs by the tPA ligand. Due to its short sequence, tPA could be a simpler ligand for target protein delivery in engineered EVs.

### 2.2 The EVs‐CasRx/gRNA System Suppresses Both Exogenous and Endogenous Gene Expression In Vitro

**Figure 3:**
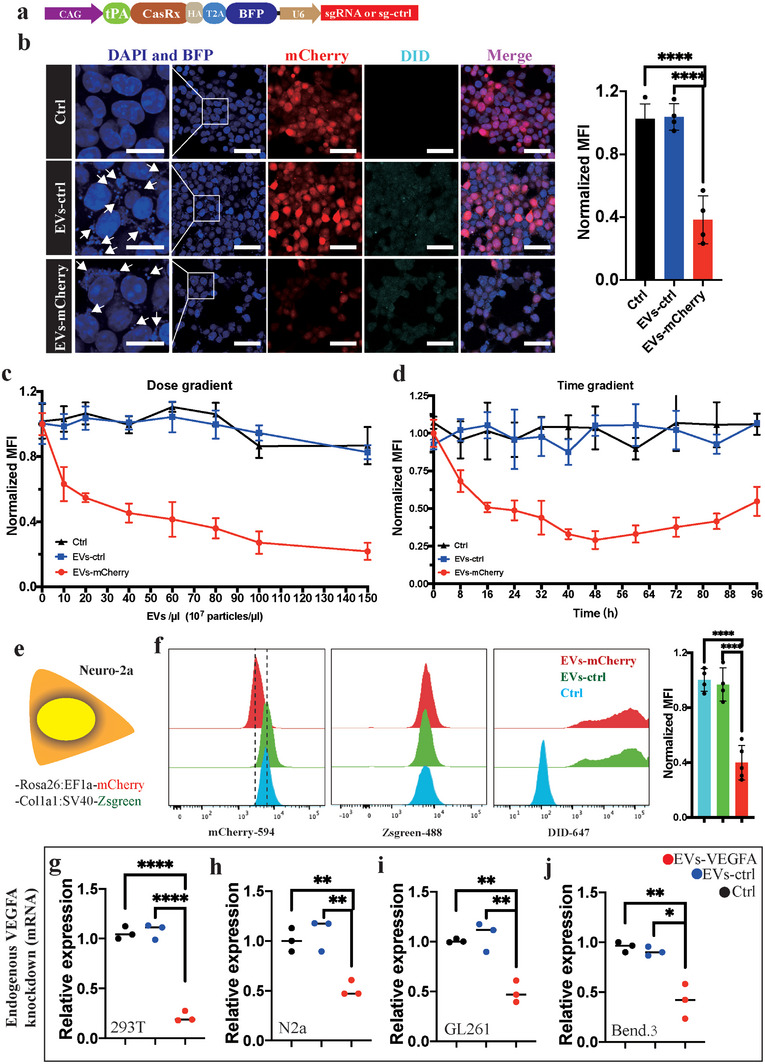
The EVs‐CasRx/gRNA system inhibits exogenous and endogenous gene expression in vitro. a) Schematic illustrating the EV plasmid design. b) Immunostaining with DAPI, BFP, mCherry, and DiD shows that DiD‐labeled EVs‐mCherry entered the target cell, leading to decreased mCherry expression. The BFP in EVs was visualized in the target cell (white arrows) (left). Scale bars = 50 µm for the right and 20 µm for the left. Statistical analysis of mean fluorescence intensity (MFI) (right). c) The change in the mean fluorescence intensity after the administration of different numbers of EVs‐mCherry particles showed that the MFI decreased as the dose increased. d) Change in MFI over time showing that the MFI decreased within 48 h but gradually returned to the original level after 48 h. e) Schematic illustration of the Neuro‐2a cell line with double fluorescence staining. f) Distribution of Neuro‐2a cells in different spectral channels showing that the distribution of the EVs‐mCherry shifted to the left in channel 594. Moreover, the DiD dye labeling EVs was visible in channel 647 (left panel). Statistical analysis of MFI (right panel). g–j) Statistical analysis of VEGFA mRNA levels in 293T, N2a, GL261, and Bend.3 cell lines. ****p < 0.0001, ***p < 0.001, **p < 0.01, and *p < 0.05. The data are presented as the means ± SD.

As a first proof‐of‐concept experiment, we constructed a HEK293T cell line stably expressing mCherry using a lentivirus. The DiD (a far‐red plasma membrane fluorescent probe)‐marked EVs carrying CasRx and gRNA targeting mCherry (namely, EVs‐mCherry) or EVs carrying CasRx and control gRNA (namely, EVs‐ctrl) were added to the medium (**Figure** **3**a). The ICC results revealed that EVs‐mCherry, but not EVs‐ctrl, significantly suppressed mCherry expression (Figure 3b). Notably, BFP was also loaded into EVs due to the T2A design (Figure 3b). Importantly, we found that EVs‐mCherry suppressed the expression of mCherry in a dose‐dependent manner by measuring the mCherry mean fluorescence intensity (Figure 3c). In contrast to viral delivery of CRISPR/Cas, EVs‐mediated delivery is instantaneous and transient. The protein delivered by EVs is catabolized and degrades rapidly after performing its function.^[^ ^5e^ ^]^ Therefore, we next sought to determine the lifespan of the delivered EVs‐mCherry by performing a time course experiment. CRISPR/Cas delivered by the virus is the nucleic acid that must be transcribed and translated before functioning, while the EVs deliver the protein, which functions immediately after it enters the target cell (Figure 3d). Before 48 h, the fluorescence intensity decreased with time, but the fluorescence intensity began to increase slowly after 48 h (Figure 3d), which revealed that the CasRx protein and/or the gRNA targeting mCherry was degraded by the target cell. Thus, delivering CasRx via EVs is a relatively short‐acting method of RNA perturbation.

We constructed a Neuro‐2a cell line expressing both mCherry and Zsgreen fluorescent proteins to examine whether these engineered EVs from human HEK293T cells would work in a murine cell line (Figure 3e). The expression of mCherry, but not Zsgreen, was markedly decreased in the EVs‐mCherry group compared with the EVs‐ctrl and no EVs control groups (Figure 3f). The cells that received EVs were confirmed by a flow cytometry analysis of the EVs‐tracking agent DiD (Figure 3f), as well as the fluorescence staining for CasRx‐HA (Figure S4a,b, Supporting Information). Additionally, the viability of HEK293T and N2a cells was not affected by engineered EVs (Figure S4c,d, Supporting Information).

Previous studies have successfully inhibited vascular endothelial growth factor A (VEGFA) expression using the CRISPR/Cas13 system.^[^ ^13^ ^]^ We further tested whether EVs delivering CasRx and gRNAs could knock down VEGFA expression in different cell lines. The double gRNAs that were capable of targeting both murine and human VEGFA were linked by a direct repeat sequence (DR) (Figure S4e, Supporting Information).^[^ ^13^ ^]^ The VEGFA mRNA levels in 293T and N2a cells decreased significantly after EVs‐VEGFA treatment (Figure 3g,h). The VEGFA mRNA levels in 293T cells decreased before 24 h, and remained at a low level until 36 h (Figure S3f, Supporting Information). The VEGFA mRNA levels increased after 36 h (Figure S4f, Supporting Information). Then, we explored EVs‐VEGFA function in GL261 and Bend.3 cell lines (Figure S4g,h, Supporting Information), and observed that EVs‐VEGFA also reduced VEGFA expression by less than half (Figure 3i,j). Based on these results, the CasRx and gRNA system delivered by EVs rapidly and transiently reduces the expression of both exogenous genes and endogenous genes.

### 2.3 The EVs‐CasRx/gRNA System Inhibits Macrophage Activation

Acute inflammatory disease is a typical and common clinical condition that often requires urgent intervention.^[^ ^14^ ^]^ Macrophages are activated and produce many cytokines after stimulation with LPS or pathogens, which is an important step in the induction and exacerbation of acute inflammatory diseases.^[^ ^14^, ^15^ ^]^ IL‐6, TNF, and IL‐1*β* are pivotal cytokines whose positive feedback is difficult to reverse in a short period.^[^ ^14^, ^16^ ^]^ We first screened gRNAs targeting several cytokines to examine whether our EVs‐CasRx/gRNA delivery system suppressed cytokine production in macrophages and subsequent macrophage M1 phenotypic polarization. Efficient and accurate gRNAs should be screened first to specifically suppress the expression of these three cytokines via CasRx‐mediated RNA editing. Because primary or cell lines of immune cells are difficult to transfect and the transfection procedure may lead to upregulation of these cytokines,^[^ ^17^ ^]^ we cloned the cDNA sequences of these three cytokines into the overexpression vector and cotransfected them with the CasRx plasmid into HEK293T cells (Figures S5 and S6, Supporting Information). We first performed a preliminary screening by linking cytokine cDNAs with enhanced green fluorescent protein (EGFP) using a T2A sequence to allow the cytokine expression levels to be measured to some extent by monitoring the fluorescence intensity of EGFP (Figure S5a,b, Supporting Information). The flow cytometry analysis revealed that some efficient gRNAs significantly reduced EGFP levels (Figure S5c, Supporting Information), and we screened three to five gRNAs that potently inhibited EGFP expression (Figure S5d–g, Supporting Information). We developed another screening system in which the cytokine cDNAs and EGFP were driven by different promoters (Figure S6a,b, Supporting Information) such that the expression of cytokines was no longer related to EGFP to confirm the efficiency of these efficient gRNAs identified in the first round of screening (Figure S6c, Supporting Information). The most efficient gRNAs targeting IL‐6, TNF and IL‐1*β* were sgIL6‐2, sgTNF‐13 and sgIL1*β*‐9, respectively (Figure S6d–g, Supporting Information). We confirmed that engineered EVs enveloped with CasRx and the aforementioned sgRNAs (EVs‐target) suppressed the expression of corresponding cytokines by delivering EVs‐ctrl or EVs‐target into Raw264.7 cells and found that the EVs‐target system remarkably reduced the IL‐6, IL‐1*β* and TNF mRNA expression levels (Figure S7a–d, Supporting Information).

**Figure 4:**
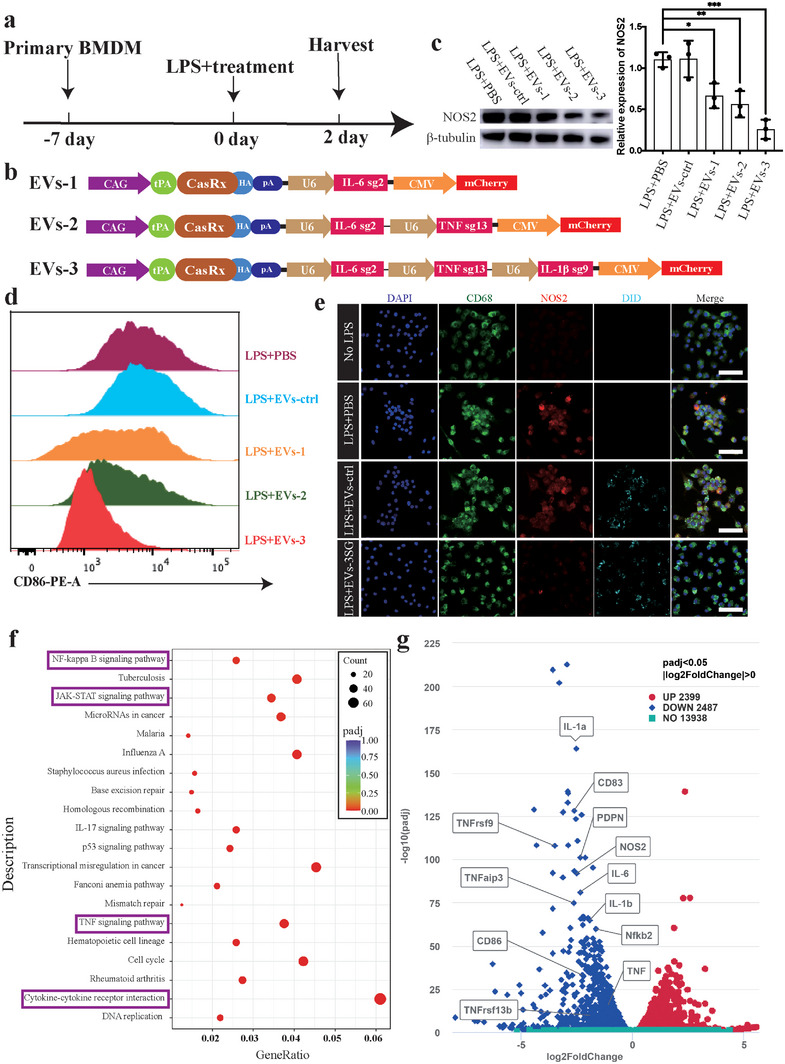
EVs‐3SG inhibit macrophage polarization. a) Schematic illustration of the experimental design used to detect the effectiveness of EVs. b) A schematic of CasRx vectors with a series of different gRNAs targeting cytokines. c) Immunoblotting results showing NOS2 expression levels (left panel). Statistical analysis of NOS2 expression based on the immunoblotting results (right panel). d) Quantification of CD86^+^ cells showing that the ability to suppress the polarization of macrophages increased from EVs‐1 to EVs‐2 and EVs‐3. e) Immunostaining with DAPI, CD68, NOS2, and DiD showing that EVs‐3SG, but not EVs‐ctrl, inhibited NOS2 expression. Scale bar = 50 µm. f) KEGG signaling pathway analysis of differentially expressed genes showing that the differentially expressed genes were enriched in the inflammatory response pathway, including the NF‐kappa B signaling pathway, JAK‐STAT signaling pathway, TNF signaling pathway and cytokine–cytokine receptor interaction pathway (purple box). g) Volcano plot of the transcriptome showing that the expression levels of typical genes associated with polarization (IL‐6, IL‐1*β*, TNF, NOS2, CD86, and NF‐*κ*B) were reduced in the EVs‐3SG‐treated group. ****p < 0.0001, ***p < 0.001, **p < 0.01, and *p < 0.05. The data are presented as the means ± SD.

To determine whether our EVs‐CasRx/gRNA system could suppress the cytokine production in macrophages and subsequent macrophage M1 phenotype polarization or not, we isolated bone marrow‐derived macrophages (BMDMs) and stimulated the BMDMs with LPS, and EVs were added simultaneously (**Figure** **4**a). We next constructed three plasmids (EVs‐1, EVs‐2, and EVs‐3) whose main components were basically the same but contained different numbers of U6‐gRNA components in the plasmids (Figure 4b). IL‐6 has always been considered the prominent cytokine causing immune hyperactivation.^[^ ^14^, ^18^ ^]^ Hence, we used the U6‐gRNA targeting IL‐6 as the cornerstone and then added IL‐1*β* and TNF*α* U6‐gRNAs (Figure 4b). After 2 d, we found that the expression of nitric oxide synthase 2 (NOS2, a marker of activated macrophages) in the EVs‐1, EVs‐2, and EVs‐3 groups was significantly lower than that in the LPS+PBS and EVs‐ctrl groups, and EVs‐3 showed the best inhibitory efficiency (Figure 4c). As an approach to verify these results, we further performed a flow cytometry assay to compare the number of CD86^+^ (a marker of M1 phenotype macrophages) macrophages. We found that the ratio of CD86^+^ cells in the EVs‐1, EVs‐2, and EVs‐3 groups was also lower than that in the no EVs and EVs‐ctrl groups, and the EVs‐3 group had the lowest number of CD86^+^ cells (Figure 4d). Moreover, we conducted ICC staining to track EVs and verify the difference in NOS2 expression between the EVs‐3 (namely, EVs‐3SG) and control groups, and the results were consistent with the immunoblotting results (Figure 4c,e). Therefore, we selected EVs‐3SG as the proper and efficient EVs for subsequent in vitro and in vivo experiments.

We repeated the aforementioned experiments in the Raw264.7 cell line and performed RNA sequencing between the EVs‐3SG and EVs‐ctrl groups to further support these findings. Kyoto Encyclopedia of Genes and Genomes (KEGG) pathway analysis was performed, and the results suggested that the NF‐kappa B signaling pathway, JAK‐STAT signaling pathway, TNF signaling pathway, and cytokine–cytokine receptor interaction pathway were downregulated in the EVs‐3SG group (Figure 4f). Notably, these four pathways are closely associated with inflammatory reactions and immune activation. The volcano plot showed that among the 2487 downregulated genes, the IL‐6, IL‐1*β*, TNF, NOS2, CD86 and NF‐*κ*B levels in the EVs‐3SG group were lower than those in the EVs‐ctrl group (Figure 4g). Thus, these results were consistent with the results obtained in BMDMs (Figure 4a–e). The aforementioned results confirmed that EVs carrying CasRx and the gRNA complex targeting cytokines inhibit macrophage polarization and attenuate the acute inflammatory response in vitro.

### 2.4 EVs‐3SG Ameliorates Lung Injury in the LPS‐Induced ALI Model

**Figure 5:**
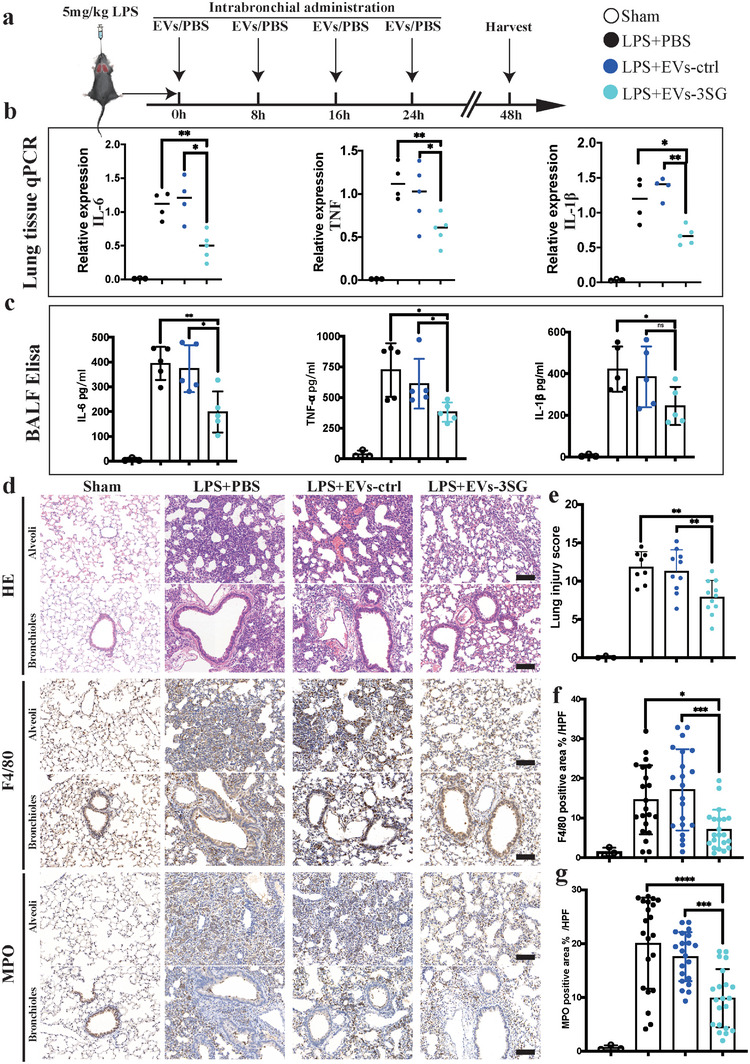
EVs‐3SG ameliorate lung injury in the LPS‐induced ALI model. a) Schematic illustrating the experimental design used for model establishment and treatment. LPS and EVs were intratracheally administered. b) Results of the statistical analysis of IL‐6, TNF, and IL‐1*β* mRNA levels in lung tissue. c) Statistical analysis of the IL‐6, TNF, and IL‐1*β* levels in BALF detected using ELISA. d) H&E and immunohistochemical staining of lung tissues from mice administered different treatments showed that EVs‐3SG reduced the immune response and alleviated lung injury. Scale bar = 100 µm. e) Statistical analysis of the pathological lung injury score. f) Statistical analysis of F4/80^+^ macrophage area % per high power filed (HPF), *n* = 10 mice per group. Two sections per mouse were analyzed. g) Statistical analysis of MPO^+^ neutrophil area % per HPF, *n* = 10 mice per group, 2 slides per mouse were analyzed. ****p < 0.0001, ***p < 0.001, **p < 0.01, and *p < 0.05. The data are presented as the means ± SD.

Acute lung injury (ALI) caused by various pathogens or trauma is characterized by acute inflammation and the upregulation of proinflammatory cytokines.^[^ ^19^ ^]^ In severe cases, ALI may cause acute respiratory distress syndrome (ARDS), a situation in which patients need assisted ventilation as soon as possible.^[^ ^20^ ^]^ Animals received a single intratracheal instillation of LPS (5 mg kg^‐1^), followed by 4 injections of EVs‐3SG to assess the therapeutic potential of EVs‐3SG in vivo (**Figure** **5**a). Then, we collected lung tissue for cytokine qPCR and found that EVs‐3SG substantially reduced IL‐6, IL‐1*β*, and TNF mRNA expression (Figure 5b). Bronchoalveolar lavage fluid (BALF) was collected, and cytokine levels in BALF were detected using ELISA, which indicated that EVs‐3SG significantly decreased the levels of these three cytokines compared with the control group (Figure 5c). The results from the lung histological examination indicated that EVs‐3SG reduced septal thickening, interstitial edema, and collagen accumulation in the bronchioles and alveoli (Figure 5d). The lung injury score was analyzed, and EVs‐3SG administration markedly ameliorated lung tissue damage (Figure 5e). We further compared the infiltration of immune cells among these different groups and found that EVs‐3SG, but not EVs‐ctrl, significantly reduced the infiltration of monocytic cells and neutrophils in both the bronchioles and alveoli (Figure 5d,f,g).

**Figure 6:**
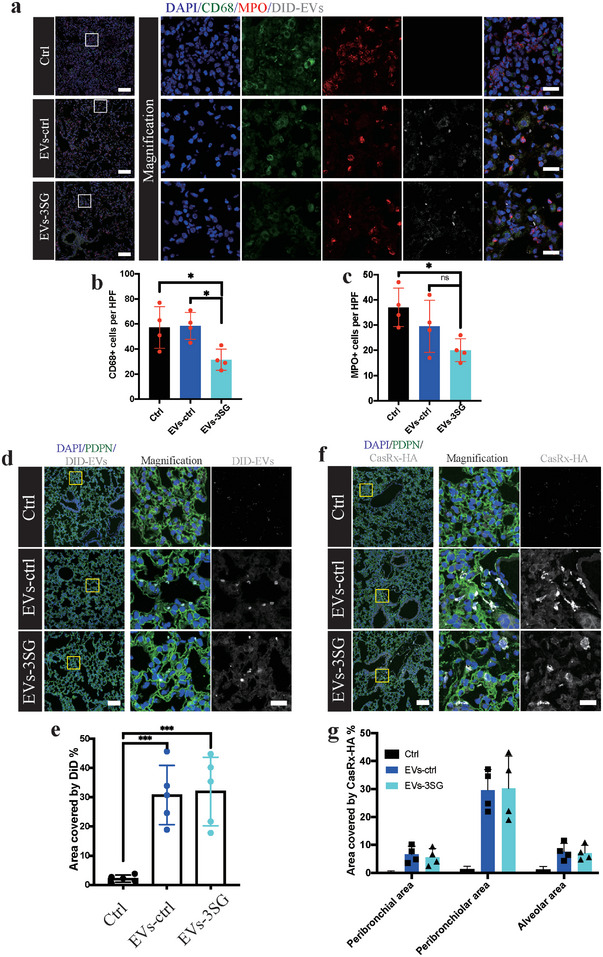
Tracking of EVs in lung tissue. a) Immunostaining for DAPI, CD68, MPO and DID revealing that EVs entered inflammatory cells. Boxed regions are magnified on the right. Scale bar = 100 µm for the left images and 20 µm for the right images. b) Quantification of CD68^+^ cells per HPF. c) Quantification of MPO+ cells per HPF. d) Immunofluorescence staining of lung tissue from mice that received different treatments. Boxed regions are magnified in the right panel. Scale bar = 100 µm for the left images, 20 µm for the right images. e) Statistical analysis of the area stained with DiD in lung tissue. f) Representative immunofluorescence images showing that visible CasRx‐HA mainly clustered around the bronchioles. Boxed regions are magnified in the right panel. Scale bar = 100 µm for the left images and 20 µm for the right images. g) Statistical analysis of the distribution of CasRx‐HA in lung tissue. ****p < 0.0001, ***p < 0.001, **p < 0.01, and *p < 0.05. The data are presented as the means ± SD.

We confirmed that EVs‐3SG entered cells in lung tissue by labeling the EVs with DiD and tracked the EVs by performing immunofluorescence staining (**Figure** **6**a). EVs were detected in macrophages (CD68^+^) and neutrophils (MPO^+^). EVs‐3SG administration efficiently decreased macrophage and neutrophil aggregation in lung tissue (Figure 6a–c). Then, we compared the tracing method of DiD and directly tracking CasRx with an HA‐tagged antibody (Figure 6d,f). DiD‐EVs were observed in pneumocytes (PDPN^+^) and covered the alveolar area and bronchiolar areas (Figure 6d,e). In contrast to the results obtained when tracking EVs with DiD, most CasRx proteins were located around the bronchioles (Figure 6f,g). One possible explanation was that these aggregates of CasRx proteins trapped in the bronchioles failed to disperse into the alveoli. On the other hand, CasRx proteins that dispersed into the alveoli were difficult to detect due to their low concentration. Therefore, compared with tracking CasRx directly, tracking EVs with DiD was more convenient and efficient. Thus, EVs‐3SG enter different lung cells after administration and ameliorate lung injury in an LPS‐induced ALI model.

### 2.5 EVs‐3SG Alleviates Organ Damage and Mortality during the Acute Phase in an LPS‐Induced Septicemia Model

**Figure 7:**
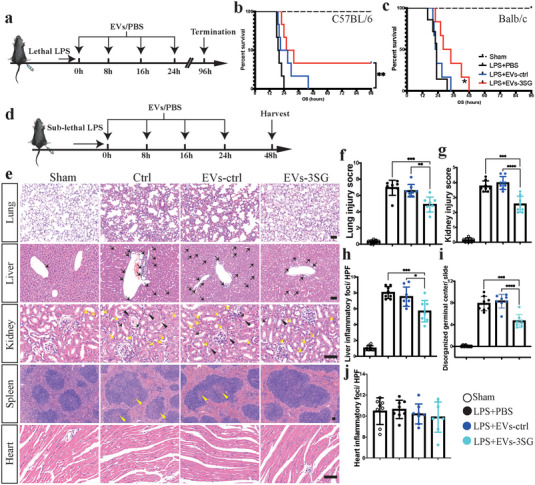
EVs‐3SG mitigate mortality and organ damage in a septicemia model. a) Schematic illustrating the experimental design used for model establishment with a lethal dose of LPS and treatment. b) Survival curve of C57BL/6 mice that received a lethal dose of LPS, *n* = 6 mice per group. c) Survival curve of BALB/c mice that received a lethal dose of LPS, *n* = 6 mice per group. d) Schematic illustrating the experimental design used for model establishment with a sublethal dose of LPS and treatment. e) H&E staining of different tissues from mice administered different treatments. EVs‐3SG significantly alleviated lung injury (line 1). In the liver, EVs‐3SG reduced the leakage of inflammatory cells (black arrows) (line 2). In addition, EVs‐3SG mitigated acute kidney injury (line 3). Inflammatory cells (yellow arrows), vacuolated renal tubules (black arrowheads), and the formation of casts (black arrows) were observed. In the spleen, the EVs‐3SG‐treated group had fewer disorganized germinal centers (yellow arrowheads) (line 4). No obvious cardiac changes were observed in the sham, ctrl, EVs‐ctrl, and EVs‐3SG groups (line 5). Scale bar = 50 µm. f) Statistical analysis of the pathological lung injury score, *n* = 8 mice per group. g) Statistical analysis of the pathological kidney injury score, *n* = 8 mice per group. h) Statistical analysis of liver inflammatory loci per HPF, *n* = 8 mice per group. i) Statistical analysis of disorganized germinal centers per HPF, *n* = 8 mice per group. j) Statistical analysis of inflammatory loci in the heart per HPF, *n* = 8 mice per group. ****p < 0.0001, ***p < 0.001, **p < 0.01, and *p < 0.05. The data are presented as the means ± SD.

Although the development and widespread use of antibiotics has reduced the incidence of sepsis, sepsis is still a life‐threatening illness.^[^ ^21^ ^]^ The systemic damage associated with sepsis is caused not only by an overwhelming pathogen load but also by severe immune activation that is not resolved in a timely manner.^[^ ^14^, ^21^ ^]^ We further questioned whether EVs‐3SG treatment would impede the severe immune response in an LPS‐induced septicemia model. A lethal dose of LPS (40 mg kg^‐1^ for C57BL/6 mice and 30 mg kg^‐1^ for BALB/c mice) was injected intraperitoneally to establish a septicemia model, followed by 4 injections of EVs‐3SG (**Figure** **7**a). Remarkably, mice that received EVs‐3SG were more resistant to LPS‐induced mortality. The C57BL/6 and BALB/c mice in the EVs‐3SG group experienced prolonged survival, and two of the C57BL/6 mice survived until the termination of the experiment (Figure 7b,c). Next, we explored the protective role of EVs‐3SG in the septicemia model by intraperitoneally injecting a sublethal dose of LPS (25 mg kg^‐1^ for C57BL/6 mice) (Figure 7d). Mouse tissues were collected 48 hours after LPS challenge, and histological examinations of the lung, liver, kidney, spleen, and heart were performed. Similar to the results obtained from the ALI model, EVs‐3SG reduced septal thickening, interstitial edema, and collagen accumulation in the lung tissue and obviously alleviated lung injury (Figure 7e,f). The tissue damage caused by the intratracheal administration of LPS was substantially more serious than that caused by the systemic administration of LPS (Figures 5b and 7e). In addition, EVs‐3SG reduced the aggregation of inflammatory cells in the liver (Figure 7e,h); however, no obvious difference was observed in the heart after the quantification of inflammatory cells (Figure 7e,j). Acute kidney injury is a common and fatal complication of sepsis.^[^ ^22^ ^]^ Compared with the PBS and EVs‐ctrl groups, EVs‐3SG alleviated damage in renal tubular cells and decreased the aggregation of inflammatory cells (Figure 7e,g). Notably, the formation of casts is an important and typical manifestation of tubular damage, and less cast formation was observed in the EVs‐3SG‐treated group than in the control and EVs‐ctrl groups (Figure 7e,g). In the LPS‐challenged spleen, many large germinal centers with unclear boundaries that were delimited and atrophic were observed (Figure 7e). Fewer disorganized germinal centers were observed in the EVs‐3SG‐treated group than in the other two control groups (Figure 7e,i).

**Figure 8:**
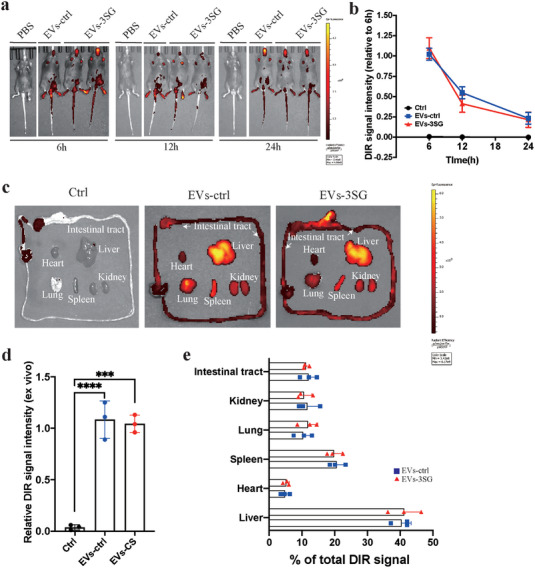
Biodistribution of systemically infused EVs detected using DiR labeling. a) In vivo imaging of PBS or DIR‐EVs at 6, 12, and 24 h after an intraperitoneal infusion. b) Relative change in the DiR signal intensity over time with in vivo imaging. c) Different organs (heart, lung, kidney, liver, spleen, and intestinal tract) were collected 24 h after the infusion of DiR‐EVs for ex vivo imaging. d) Relative DiR signal intensity detected using ex vivo imaging. e) Absolute DiR signal intensity in different organs of mice that received EVs‐ctrl and EVs‐3SG treatment. ****p < 0.0001, ***p < 0.001, **p < 0.01, and *p < 0.05. The data are presented as the means ± SD.

To determine the biodistribution of EVs, we systemically tracked EVs with DiR (a near infrared plasma membrane fluorescent probe) by in vivo imaging^[^ ^23^ ^]^ (**Figure** **8**a). The results suggested that one dose of EVs was maintained for longer than 24 h after administration before being discharged (Figure 8a,b). The heart, lung, spleen, liver, kidney, and intestinal tract were imaged ex vivo to further illustrate the organic biodistribution of EVs due to the low penetrating power of DiR (Figure 8c). The ex vivo signal intensity of mice that received EVs‐ctrl and EVs‐3SG treatments was substantially stronger than that of mice that received the PBS + DiR treatment (Figure 8d). The results suggested that approximately 60% of EVs are aggregated in the liver and spleen (Figure 8c,e). Approximately 10% of EVs were distributed in the lung, kidney, and intestinal tract (Figure 8e). Thus, engineered EVs are injected intraperitoneally to treat sepsis spread throughout the body. We also tracked EVs with DiD by performing immunofluorescence staining, and the results suggested that these EVs were distributed mainly in the lung, kidney, and particularly the liver and spleen (Figure S8, Supporting Information), consistent with the results from in vivo imaging. Based on these results, intraperitoneally injected EVs‐3SG are distributed throughout the body and markedly alleviate systemic organ damage during the acute phase in an LPS‐induced septicemia model.

## 3 Discussion

Due to the low immunogenicity and mobility of EVs, many studies have shown that EVs are efficient and ideal vehicles for delivering target DNA, RNA, and proteins.^[^ ^5^, ^24^ ^]^ Using EVs to deliver CRISPR/Cas is a promising approach to expand the application of gene editing. Furthermore, Cas9 and target gRNA were engineered and loaded into EVs to induce exon skipping and successfully improve Duchenne muscular dystrophy (DMD) symptoms in a mouse model.^[^ ^5e^ ^]^ However, Cas9 is not sufficiently safe because it induces double‐strand breaks and may lead to frameshift mutations.^[^ ^25^ ^]^ CasRx, which targets mRNA rather than DNA, appears to be a better tool to temporarily suppress the expression of target genes, as no changes to the genome are introduced.^[^ ^6a^ ^]^ Here, we developed CasRx‐loaded EVs and optimized the packaging strategy using a short tPA ligand, which can be employed to deliver a variety of proteins. We provided immunoblotting, IEM, and immunofluorescence staining data showing that the tPA ligand substantially increased the amount of the target protein in EVs. Although the specific mechanism of the tPA‐guided packaging was not explored here, a recent study of lysosome‐associated membrane protein 2, isoform A (LAMP2A) has provided some insights.^[^ ^26^ ^]^ In this study, Ferreira et al. identified a new exosome mechanism independent of the endosomal sorting complex required for transport (ESCRT) machinery, while tagging proteins with the KFERQ motif facilitated the loading of the target protein.^[^ ^26^ ^]^ We will reveal the relevant mechanisms of the tPA ligand in a subsequent study.

As a proof‐of‐concept experiment, we targeted an exogenous fluorescent protein and inhibited the expression of mCherry. In both the HEK293T (human) and Neuro‐2a (mouse) cell lines, EVs‐mCherry precisely suppressed mCherry expression. Then, we successfully knocked down endogenous VEGFA expression in different cell lines using engineered EVs. Because the CasRx delivered by EVs is a protein rather than DNA (delivered by a virus) or mRNA (delivered by lipid nanoparticles), it performs its suppressive function immediately after entering the target cells. Notably, these engineered EVs were rapidly catabolized after 48 h, indicating that they are ideal short‐acting tools to transiently suppress the expression of target genes.

**Figure 9:**
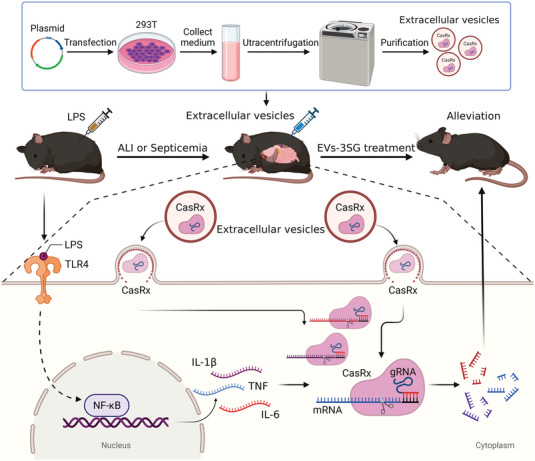
Schematic illustrating the engineered EVs‐delivered CRISPR/CasRx RNA editing tool. The engineered EVs‐delivered CasRx/gRNA system degrades TNF, IL‐1*β* and IL‐6 mRNAs to relieve the cytokine storm triggered by LPS, thereby alleviating acute inflammatory diseases in mice.

The CRISPR/Cas system has been applied in inherited diseases and neurodegenerative diseases.^[^ ^7^, ^13^, ^27^ ^]^ However, few CRISPR systems are available for acute diseases, as represented by acute inflammatory diseases.^[^ ^28^ ^]^ Under normal conditions, cytokines are produced and catabolized rapidly due to their short half‐life.^[^ ^29^ ^]^ However, in certain aggressive inflammatory disease, cytokines are excessively produced, and the immune response fails to reduce their levels, inevitably leading to the development of a cytokine storm due to the positive feedback of various cytokines.^[^ ^14^, ^16^ ^]^ Excess cytokines and immune activation interact and form a vicious cycle. TNF and IL‐1*β* increase vascular permeability, and IL‐6 induces complement expression to promote innate immune activation^[^ ^30^ ^]^ These activated immune cells produce more cytokines, exacerbating the systemic damage. Currently, the typical conditions that involve a cytokine storm are COVID‐19 and cytokine release syndrome resulting from chimeric antigen receptor T‐cell (CAR‐T) therapy.^[^ ^31^ ^]^ Considering these circumstances, we screened out the most efficient gRNAs targeting IL‐6, IL‐1*β*, and TNF by two‐step strategies. This EV system containing the CasRx/gRNA complex can transiently knock down any gene via the above strategy. We further verified that EVs‐3SG suppressed the proinflammatory response of primary macrophages and monocytic cell lines. Moreover, our in vivo data supported the conclusion that EVs‐3SG ameliorates the damage to different organs caused by proinflammatory cytokines and immune cells. The biodistribution of engineered EVs was illustrated by performing in vivo imaging and immunofluorescence staining, which confirmed that one dose of intraperitoneally administered EVs was distributed in all important organs and persisted for longer than 24 h. Our study could be summarized by an illustration (**Figure** **9**). This system may be extended to a variety of acute diseases, such as stroke, acute kidney disease, and acute myocardial infarction, and is also suitable for the acute control of certain autoimmune diseases.

This study also has some limitations. We did not reveal the specific mechanism by which the tPA ligand loaded certain proteins into EVs, which should be elucidated in a future study. The multistep reactions required to obtain engineered EVs may be time consuming. CasRx and gRNA complexes are constantly catabolized in target cells; thus, a large number of EVs are necessary to ensure the efficacy of the treatment. In follow‐up studies, the optimization of loading of U6‐gRNAs^[^ ^5e^ ^]^ should be explored. Moreover, improving EV targeting is also an urgent problem to be solved.^[^ ^32^ ^]^

In conclusion, we developed a promising tool for EV delivery of CRISPR/CasRx and gRNA complexes to rapidly and transiently perturb the expression of target genes. We verified the function of this system in ALI and septicemia models, which revealed good therapeutic outcomes in the acute phase. Further explorations of the use of this approach for other acute inflammatory diseases (e.g., CRS caused by CAR‐T therapy and autoinflammatory disorders) are worthwhile. More importantly, we provide a novel method to temporarily regulate the expression of target genes and apply gene editing for the treatment of acute diseases.

## 4 Experimental Section

### Animals

All animal experiments were performed in accordance with standard guidelines for the care and use of laboratory animals. All animal experiments were approved by the Institutional Animal Care and Use Committee of Fudan University (approval number, 2022080013Z). C57BL/6 and BALB/c mice were purchased from Shanghai SLAC Laboratory Animal Co., Ltd. (Shanghai, China).

### Extraction and Storage of EVs

To produce the engineered EVs, 293T cells in good condition were seeded into 15 cm dishes with complete medium (DMEM + 10% fetal bovine serum + 1% penicillin‐streptomycin + 1% non‐essential amino acids). When the cell density reached 70–80%, the 293T cells were transfected with the corresponding plasmids (50 µg per dish. All plasmids (DNA) have a certain mass, which can be measured by instruments such as Nanodrop. In general, the measured unit of plasmid mass is ng µL^‐1^.50 µg (50 000 ng) plasmids were typically transfected into a 15 cm dish HEK293T cells. Therefore, 50 µg (50 000 ng) was divided by concentration (ng µL^‐1^) to obtain the volume, and quantitative transfection can be achieved) with EZ Trans Cell Transfection Reagent (AC04L098, Life iLab Bio Co., Ltd.) according to the manufacturer's instructions. After 24 h, the medium was carefully removed, the cells were rinsed with PBS, and medium without FBS (DMEM + 1% penicillin–streptomycin + 1% nonessential amino acids) was added. After 24–48 h, the cell medium was harvested. The medium was subjected to the following centrifugation steps: 1) 300 × *g* for 10 min to remove cells; 2) 2000 × *g* for 10 min to remove dead cells; 3) 10 000 × *g* for 30 min to remove cell debris; and (4) 150 000 × *g* for 70 min for ultracentrifugation of EVs at 4 °C (Ultracentrifuge, Optima L‐90K, Beckman; Centrifuge bottles, 355618, Beckman). Then, the EVs were washed and resuspended in PBS, followed by another ultracentrifugation step at 150 000 × *g* for 70 min.

The EVs should be stored at 4 °C for short‐term storage (<3 d). For long‐term storage (>3 d), the EVs should be stored at ‐80 °C. Using them immediately after extraction and purification instead of after long‐term storage is strongly suggested. Fresh EVs are more effective than freeze‐thawed EVs.

### Tracking EVs with DiD

Fluorescent labeling of the collected EVs with DiD was performed according to the manufacturer's instructions (C1039, Beyotime), and DiD was added to the EVs at a final concentration of 10 × 10^‐6^ m. The unconjugated fluorescent dye was removed by washes through a 0.22 µm filter membrane (FF372, Beyotime).

### TEM and IEM

EVs were evaluated morphologically by performing negative staining. First, 10 µL of EVs suspended in PBS were loaded onto glow‐discharged carbon‐coated copper grids (T11023, Beijing XXBR Technology Co., Ltd). After sample adsorption for 1 min, the grid was blotted with filter paper and stained with 2% uranyl acetate for 2 min. Next, the samples were dried for 20 s using a dryer. EVs were viewed under an electron microscope (Talos L120C, Thermo Fisher, USA) at a voltage of 120 kV.

IEM was performed as described previously.^[^ ^12a^ ^]^ For immunogold labeling with antibodies, EVs were fixed with 2.5% PFA for 30 min, washed twice with PBS, dissolved in PBS/0.5% BSA, deposited onto formvar carbon‐coated electron microscopy grids (T11023N, Beijing XXBR Technology Co., Ltd.), and exposed for 10 min in a dry environment. Then, EVs on the grids were washed five times (3 min each) with PBS/0.5% BSA. Fixed EVs on the grid were incubated with 5% BSA for 30 min at room temperature, washed five times with PBS/0.5% BSA (3 min), transferred to a drop of antibody (1:50 dilution for the anti‐CD63 antibody, Cat# ab134045, and anti‐HA tag antibody, Cat# ab9110) in PBS/0.5% BSA, and incubated for 2 h at room temperature. Afterward, EVs on the grids were washed five times with PBS/0.5% BSA (3 min), incubated with preabsorbed goat anti‐rabbit IgG H&L Gold (10 nm) (Abcam, Cat#ab39601) in PBS/0.5% BSA for 1 h at room temperature, and then washed five times (3 min) with PBS/0.5% BSA. Finally, EVs on the grids were stained with 2% uranyl acetate and then viewed under an electron microscope.

### Analysis of EVs by NTA, Zeta Potential, and Polydispersity Index

The EV particle size and concentration were using NTA at VivaCell Shanghai with ZetaView PMX 110 (Particle Metrix, Meerbusch, Germany) and the corresponding software ZetaView 8.04.02. The zeta potential and polydispersity index analysis of EVs were measured with a Malvern Zetasizer Nano ZS90 (United Kingdom). Isolated EV samples were appropriately diluted with 1× PBS buffer (Biological Industries, Israel) to measure the particle size and concentration. NTA measurements were recorded and analyzed at 11 positions. The ZetaView system was calibrated using 110 nm polystyrene particles. The temperature was maintained at approximately 23 °C and 30 °C.^[^ ^33^ ^]^ The final raw data for each sample were imported into GraphPad Prism software (version 8.3.1) for further analysis and comparison.

### Ligand Sequence, gRNA Sequence, and Plasmid Resource

The sequences of the ARRDC1 and WW domains were described previously.^[^ ^5d^ ^]^ The sequences of tPA, mouse Ig heavy chain, and human insulin are listed below:

tPA: MDAMKRGLCCVLLLCGAVFVSP

Mouse Ig heavy chain: MGWSCIILFLVATATGVHS

Human insulin: MALWMRLLPLLALLALWGPDPAAA

The gRNA sequences are listed in Table S1 (Supporting Information). The cytokine sequences were cloned from the cDNAs of LPS‐activated Raw264.7 cells to construct the “CAG‐cytokine‐T2A‐EGFP” and “CAG‐cytokine‐EF1a‐EGFP” vectors. All the plasmids were confirmed to be correct by sequencing (Biosune Biotechnology, Shanghai).

### LPS‐Induced ARDS Model

Eight to ten week old male C57BL/6 mice were used in the experiments. After anesthetization, the mice received an intratracheal instillation of a nonlethal dose (5 mg kg^‐1^) of LPS from *Escherichia coli* O111:B4 (Sigma–Aldrich, St. Louis, MO, USA) dissolved in 60 µL of PBS to establish an ARDS model. After this procedure, 3.0 × 10^11^ particles of EVs‐3SG or EVs‐ctrl were administered to mice via intratracheal instillation at 0, 8, 16, and 24 h. As a control, an equivalent volume of PBS was instilled in the same manner.

### LPS‐Induced Lethal Endotoxemia Model and Sublethal Endotoxemia Model

Eight‐ to ten week old male C57BL/6 or BALB/c mice were used in the experiments. LPS from *E. coli* O111:B4 dissolved in 300 µL of PBS was injected intraperitoneally at 40 mg kg^‐1^ (C57BL/6) or 30 mg kg^‐1^ (Balb/c). For sublethal endotoxemia model, LPS from *E. coli* O111:B4 dissolved in 300 µL of PBS was injected intraperitoneally at 20 mg kg^‐1^. After this procedure, 1.0 × 10^12^ particles of EVs‐3SG or EVs‐ctrl were administered to mice via intraperitoneal injection at 0, 8, 16, and 24 h. As a control, an equivalent volume of PBS was instilled in the same manner. The survival of the mice was evaluated every hour during the initial 48 h. Heart, lung, liver, spleen, and kidney samples were collected for further analysis.

### Cell Culture, Cell Stimulation, and RNA Sequencing

The Neuro‐2a (Rosa26:EF1a‐mCherry; Col1a1:SV40‐Zsgreen) cell line was a gift from Ni Gao. Mouse monocytes (Raw264.7) and the L929 cell line were purchased from the FuHeng Cell Center (FH0328 and FH0534, Shanghai, China) and cultured in DMEM containing 10% fetal bovine serum (FBS; Gibco, USA) and 1% penicillin/streptomycin (P/S; Thermo Fisher Scientific). Bone marrow‐derived macrophages (BMDMs) were obtained as described previously.^[^ ^34^ ^]^ Briefly, primary cells were harvested from the femur and tibia bones of C57BL/6 mice. After removing red blood cells by adding ACK lysis buffer, the cells were resuspended in DMEM containing 10% FBS, 20% L929 conditioned medium, and 1% P/S and then plated in 24‐well plates or chambers. All cell lines tested negative for mycoplasma contamination by qPCR.

Then, LPS was added to the medium of BMDMs or Raw264.7 cells at a final concentration of 100 ng mL^‐1^ and cultured for 24 h to fully activate the cells. At the same time, 8.0 × 10^10^ particles of the appropriate EVs (EVs‐ctrl or EVs‐3SG) were added to the EV groups. As a control, an equivalent volume of PBS was added in the same manner. Polarized cells were harvested for subsequent reverse transcription‐quantitative polymerase chain reaction (RT‐qPCR) analysis, flow cytometry analysis, immunoblotting, RNA‐seq, and ICC staining.

RNA‐seq of LPS‐stimulated Raw264.7 cells was performed by Novogene Bioinformati3SG Technology Co., Ltd., Beijing, China. Genes were defined as differentially expressed when their logarithmic expression ratio showed a difference of more than twofold (*p < 0.05). The mRNA level was defined as differentially expressed when the logarithmic expression ratios showed a difference >1.5‐fold (*p < 0.05).

### Hematoxylin and Eosin (H&E) Staining

For H&E staining of the lung, liver, kidney, spleen, and heart, mice were perfused with 4% paraformaldehyde (PFA) to collect tissues, which were then washed with PBS to remove excess blood. The fresh tissue was fixed with a liquid fixative for more than 24 h. Then, paraffin sections of each organ were prepared. The tissue was removed from the liquid fixative, and the target tissue was trimmed with a scalpel in a ventilated cupboard. The trimmed tissue and the label were placed in a dehydration box. Dehydration and deparaffinization were performed as described below. The dehydration box was placed into the dehydrator for dehydration with a gradient of alcohol solutions: 75% alcohol for 4 h, 85% alcohol for 2 h, 90% alcohol for 2 h, 95% alcohol for 1 h, anhydrous ethanol I for 30 min, anhydrous ethanol II for 30 min, alcohol benzene for 5–10 min, and xylene II for 5–10 min. Then, the samples were placed at 65 °C for 1 h to melt paraffin in step I, 65 °C for 1 h to melt paraffin in step II, and 65 °C for 1 h to melt paraffin in step III. The paraffin‐soaked tissue was embedded in the embedding machine. First, the melted paraffin was placed into the embedding frame, and before the paraffin solidified, the tissue was removed from the dehydration box and placed into the embedding frame according to the requirements of the embedding surface, affixing the corresponding label. The samples were cooled at ‐20 °C on a freezing table, and after the paraffin was solidified, the paraffin block was removed from the embedding frame and trimmed. The trimmed paraffin block was cooled at ‐20 °C on a freezing table, and the modified paraffin‐embedded tissue sample was sliced using a paraffin slicer at a slice thickness was 4 µm. The tissue was flattened by floating the slice in 40 °C warm water in the spreading machine, and the tissue was placed on a glass slide and baked in the oven at 60 °C. After the oven‐baked, dried paraffin was melted, it was removed and stored at room temperature. Dewaxing was performed as follows: xylene I for 20 min; xylene II for 20 min; 100% ethanol I for 5 min; 100% ethanol II for 5 min; 75% ethanol for 5 min; and a rinse with tap water. The sections were stained with a hematoxylin solution for 3–5 min and rinsed with tap water. Then, the sections were treated with a hematoxylin differentiation solution and rinsed with tap water. The sections were treated with Scott's tap water substitute as a bluing reagent, rinsed with tap water, and treated with 85% ethanol for 5 min and 95% ethanol for 5 min. Finally, the sections were stained with eosin for 5 min. The sections were dehydrated as follows: 100% ethanol I for 5 min; 100% ethanol II for 5 min; 100% ethanol III for 5 min; xylene I for 5 min; and xylene II for 5 min. Then, the sections were sealed with neutral gum. Finally, the sections were observed under a microscope. 3D View (version 2.2.0) was used for image acquisition and analysis.

### Immunofluorescence Staining and ICC Staining

For immunofluorescence staining of the lung, mice were perfused with 4% paraformaldehyde (PFA) to collect cells or tissues, which were then washed with PBS to remove excess blood. Then, the tissue was embedded in paraffin (described in detail in the H&E staining section). Sections were washed three times with PBS (pH 7.4) using a Rocker device for 5 min each. Then, 3% BSA was added to cover the tissue to block nonspecific binding and incubated for 30 min. The blocking solution was carefully removed. The slides were incubated with the primary antibody (diluted with PBS appropriately) overnight at 4 °C and placed in a humid chamber containing a small amount of water. The slides were washed three times with PBS (pH 7.4) using a Rocker device for 5 min each. Then, the liquid was carefully removed. The tissue was immersed in a secondary antibody (appropriate for recognition of the primary antibody) and incubated at room temperature for 50 min in the dark. The samples were washed three times with PBS (pH 7.4) using a Rocker device for 5 min each. Then, the cells were incubated with the DAPI solution at room temperature for 10 min in the dark. The samples were washed three times with PBS (pH 7.4) using a Rocker device for 5 min each. The liquid was carefully removed, and the sections were coverslipped with an antifade mounting medium.

For immunofluorescence staining, the cells were washed three times with PBS to exclude debris and fixed with 4% PFA. The slides were air‐dried for 1 h at room temperature and then blocked with BSA for 45 min. Primary antibodies were applied, and the sections were incubated overnight at 4 °C, followed by extensive washes with PBS to remove unbound primary antibodies. Colors were developed with secondary antibodies for 2 h at room temperature. Finally, the sections were washed three times in PBS and coverslipped with an antifade mounting medium.

As a method to track EVs in different organs, mice were euthanized 24 h after model establishment and then perfused with 4% PFA to collect target tissues, which were then washed in PBS to remove excess blood. The tissues were embedded in an optimal cutting temperature (OCT) compound and cut into 8–10 µm cryosections on a cryostat. DAPI (2 µg mL^‐1^) was added to stain the nuclei of the cells, and the cells were washed 3 times with PBS. Then, the coverslips were mounted with antifade mounting medium.

Sections labeled with fluorescence reporters were observed and photographed using an Olympus FV3000 confocal microscope. Fluorescence images were analyzed with ImageJ software (NIH). The primary and secondary antibodies and dilutions used in this study were as follows:

Rat anti‐PDPN, 1:1000, Abcam Cat#ab256559

Rabbit anti‐CD68, 1:1000, Abcam Cat#ab125212

Mouse anti‐MPO, 1:1000, Servicebio Cat# GB12224

Rabbit anti‐HA‐tag, 1:500, Abcam Cat#ab9110

Mouse anti‐NOS2, 1:500, Santa Cruz Cat#sc‐7271

Mouse anti‐CD63, 1:500, Abcam Cat#ab271286

Rabbit anti‐GFAP, 1:200, Proteintech Cat#16825

Mouse anti‐CD31, 1:500, Abcam Cat#24590

Donkey anti‐rabbit IgG Alexa Fluor 488, 1:500, Jackson ImmunoResearch Labs Cat# 711‐545‐152

Donkey anti‐mouse IgG Alexa Fluor 488, 1:500, Jackson ImmunoResearch Labs Cat# 715‐545‐150

Donkey anti‐rabbit IgG (H+L) Cy5, 1:500, Jackson ImmunoResearch Labs Cat#711‐175‐152

Donkey anti‐Rat IgG Alexa Fluor 488, 1:500, Jackson ImmunoResearch Labs Cat#711‐165‐152

Goat anti‐mouse IgG Alexa Fluor 594, 1:500, Abcam Cat# 150116

### In Vivo and Ex Vivo Imaging of DIR‐Marked EVs

EVs for biodistribution assays were labeled with the near‐infrared fluorescent dye DiR (MX4005, Shanghai Maokangbio Biotechnology Co., Ltd.). Then, 1.0 × 10^12^ particles of DiR‐labeled EVs were injected intraperitoneally and the in vivo imaging was conducted at 6, 12, and 24 h. The organic distribution of EVs was determined by intraperitoneally injecting 1.0 × 10^12^ particles of DiR‐labeled EVs and performing ex vivo imaging at 6 h. In vivo and ex vivo imaging was conducted using a Vevo 2100 Imaging System (Fujifilm, Japan), and images were analyzed using Living Image software.

### BALF Collection

BALF was harvested via the injection and retraction of 1 mL of PBS‐EDTA three times. The cells that had been pelleted from the BALF were resuspended in double‐distilled water with ACK lysis buffer and then washed twice with PBS. Fresh BALF was used for ELISA.

### Measurement of Inflammatory Cytokines and HA‐Tag by ELISA

The levels of tumor necrosis factor‐*α* (TNF‐*α*, Product# ml002095‐2), interleukin‐1*β* (IL‐1*β*, Product# ml063132‐2), and interleukin‐6 (IL‐6, Product# ml002293‐2) in BALF were detected with ELISA kits in accordance with the manufacturer's instructions (Shanghai Enzyme‐linked Biotechnology Co., Ltd.). The levels of HA‐tagged proteins in EVs were detected with ELISA kits (Human HA, Product# YJ550409, Shanghai Yuanjie Biotechnology Co., Ltd.). The EV lysis solution was used for the detection of dissociated HA‐tags (Product# 41211ES20, Shanghai Yeasen Biotechnology Co., Ltd.). The absorbance was read at 450 nm.

### Fluorescence‐Activated Cell Sorting and Analysis

HEK293T cells were exposed to lentivirus (Ubi‐Flag‐SV40‐mCherry‐IRES‐puro) for 24 h to obtain cells stably expressing mCherry. After 48 h, single mCherry+ cells were sorted in 96‐well plates using a MoFlo Astrios EQ instrument (Beckman).

HEK293T cells were transfected with the pair of plasmids shown in Figure S5b (Supporting Information) to identify potentially efficient gRNAs targeting different cytokines. The cells were collected and analyzed 48 h after transfection using an LSRFortessaX‐20 instrument (BD Bioscience).

For the establishment of the dose gradient curve, mCherry+ HEK293T cells were seeded in 24‐well plates, and fluorescence intensity was detected 48 h after adding different doses (10, 20, 40, 60, 80, 100, and 150 µL) of standard EVs‐ctrl, EVs‐mCherry, or PBS with an LSRFortessaX‐20 instrument (BD Bioscience). Three replicates were analyzed for each dose.

For the establishment of the time curve, mCherry+ HEK293T cells were seeded in 24‐well plates, and 80 µL of standard EVs were added to the medium. The fluorescence intensity was detected at 0, 8, 16, 24, 32, 40, 48, 60, 72, 84, and 96 h. Three replicates were analyzed at each time point.

The mCherry intensity in Neuro‐2a cells (Rosa26:EF1a‐mCherry; Col1a1:SV40‐Zsgreen) was detected by adding 8.0 × 10^10^ particles of DiD‐labeled EVs or PBS to the medium. After 48 h, the cells were collected and analyzed with an LSRFortessaX‐20 instrument (BD Bioscience).

For the flow cytometry analysis of LPS‐stimulated BMDMs, BMDMs were digested with 0.25% trypsin‐EDTA and resuspended in staining buffer (SB). Blocking agent (0.25 µg per 10^6^ cells) was added (BioLegend, TruStain FcXPLUS, Cat# 156603), followed by an incubation with the primary antibodies (BioLegend, CD86, Cat# 105007) for 30 min on ice. The samples were washed with SB three times before testing using an LSRFortessaX‐20 (BD Bioscience).

All flow cytometry data were analyzed with FlowJo (v10.4) software (TreeStar).

### Evaluation of the Organ Injury Scores

The details of the lung injury score have been described previously.^[^ ^35^ ^]^ Briefly, 80 high‐power fields (HPFs) in microscopy images of tissues from each mouse were examined and analyzed to calculate the final lung injury score.

The details of the kidney injury score have been described previously.^[^ ^12b^ ^]^ Briefly, 60 HPFs in microscopy images of tissues from each mouse were examined and analyzed to calculate the final kidney injury score. All evaluations were conducted by two pathologists in a blinded manner.

### Quantitative RT‐PCR

The cells or tissues were lysed using TRIzol reagent (Invitrogen). RNA was extracted with TRIzol according to the manufacturer's instructions (Invitrogen), and the RNA was converted to cDNA using HiScript III RT SuperMix (Vazyme). SYBR Green qPCR master mix (Applied Biosystems) was used, and cDNAs were amplified on a Roche LC 480 II real‐time PCR system (Roche). The sequences for the IL‐6, TNF, IL‐1*β*, and VEGFA primers are listed in Table S2 (Supporting Information).

### Immunoblotting Analysis

BMDMs from different groups were treated with various concentrations of each tested compound for the designated time. Then, the cells were lysed using 1× SEMS sample lysis buffer containing protease and phosphatase inhibitors. Cell lysates were loaded into 8–12% SEMS‐PAGE gels and electrophoresed, and then the separated proteins were transferred to PVDF membranes, which were blocked with 5% fat‐free milk in TBS solution containing 0.5% Tween‐20 for 4 h at room temperature. Then, the membranes were incubated with the NOS2 antibody (1:500, Cat# 690902, BioLegend) overnight at 4 °C, followed by washing with TBST and incubation with an HRP‐conjugated secondary antibody (1:2000, Cat# 115‐035‐003, Jackson ImmunoResearch) for 2 h. The protein signals were visualized with an ECL Western blotting detection kit (Thermo Scientific, Waltham, MA, USA). This experiment was repeated three times independently.

For the EV analysis, EVs from different groups were collected. The procedure was identical to that described above. Antibodies against the following proteins were used: CD63 (25682‐1‐AP, Proteintech), TSG101 (28283‐1‐AP, Proteintech), HA‐tag (ab9110, Abcam), and *β*‐actin (66009‐1‐Ig, Proteintech). All WB experiments were repeated three times independently.

### Statistical Analysis

The data are presented as the means ± SD. Cell counting and quantification of Western blots were performed using ImageJ software. The mean fluorescence intensity and other flow cytometry analyses were analyzed using FlowJo software (ver. 10.4). Comparison between two groups was analyzed by unpaired two‐tailed Student's *t*‐test and comparison among more than two groups was analyzed by one‐way ANOVA test using GraphPad Prism software (ver. 8.0). *P < 0.05, **P < 0.01, ***P < 0.001, and ****P < 0.0001 were considered statistically significant. All data were obtained from several independent experiments, as indicated in each figure legend. No statistical method was used to predetermine the sample size.

## Conflict of Interest

The authors declare no conflict of interest.

## Author Contributions

T.L., L.Z., T.L., and T.Z. contributed equally to this work. T.L. conceptualized the idea and carried out cell experiments, animal experiments, data analysis, and paper writing. L.Z. performed experiments, data analysis, and paper writing. T.L. carried out EVs extraction and purification. T.Z. carried out the data analysis and figure integration. N.G., C.F., and J.Y. constructed plasmids. F.L., K.C., Q.T., Q.Z., and J.R. were responsible for pathological diagnosis and preparation of paraffin sections. J.Z., J.Z., and X.D. were responsible for frozen tissue processing and immunofluorescent staining. H.Z. designed the research project. J.Z. supervised the project and wrote the paper.

## Supporting information

Supporting Information

## Data Availability

The data that support the findings of this study are available from the corresponding author upon reasonable request.
