# Supplementary material for: Engineered Extracellular Vesicle‐Delivered CRISPR/CasRx as a Novel RNA Editing Tool
Source: Adv Sci (Weinh). 2023 Feb 2;10(10):2206517. doi: 10.1002/advs.202206517 (PMC10074121; doi:10.1002/advs.202206517)
Supplement: Supplementary file 1 — Supporting Information [file ADVS-10-2206517-s001.pdf]

ADVANCED SCIENCE
Open Access

Supporting Information

for *Adv. Sci.*, DOI 10.1002/advs.202206517

Engineered Extracellular Vesicle-Delivered CRISPR/CasRx as a Novel RNA Editing Tool

Tianwen Li, Liansheng Zhang, Tao Lu, Tongming Zhu, Canbin Feng, Ni Gao, Fei Liu, Jingyu Yu, Kezhu Chen, Junjie Zhong, Qisheng Tang, Quan Zhang, Xiangyang Deng, Junwei Ren, Jun Zeng, Haibo Zhou and Jianhong Zhu**

Supplementary Materials

Engineered extracellular vesicle-delivered CRISPR/CasRx as a novel RNA editing tool

Tianwen Li[1,4], Liansheng Zhang[2,4], Tao Lu[2,4], Tongming Zhu[1,4], Canbin Feng[2], Ni Gao[2], Fei Liu[3], Jingyu Yu[1], Kezhu Chen[1], Junjie Zhong[1], Qisheng Tang[1], Quan Zhang[1], Xiangyang Deng[1], Junwei Ren[1], Jun Zeng[1], Haibo Zhou[2,*], Jianhong Zhu[1,*]

Correspondence: hbzhou@ion.ac.cn; jzhu@fudan.edu.cn

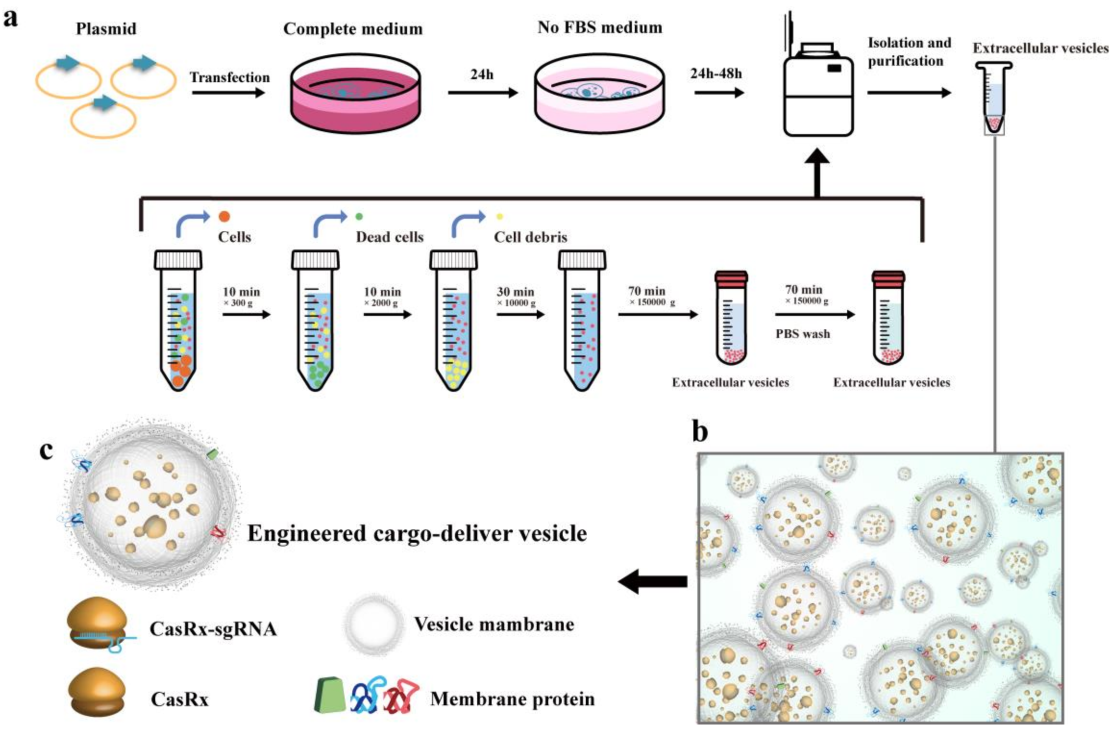

Supplemental Fig. 1 | Schematic illustration of the extraction and purification of engineered EVs. a, Procedures used to extract and purify engineered EVs. **b,** Schematic illustration of engineered EVs. **c**. Schematic illustration of the components of engineered EVs.

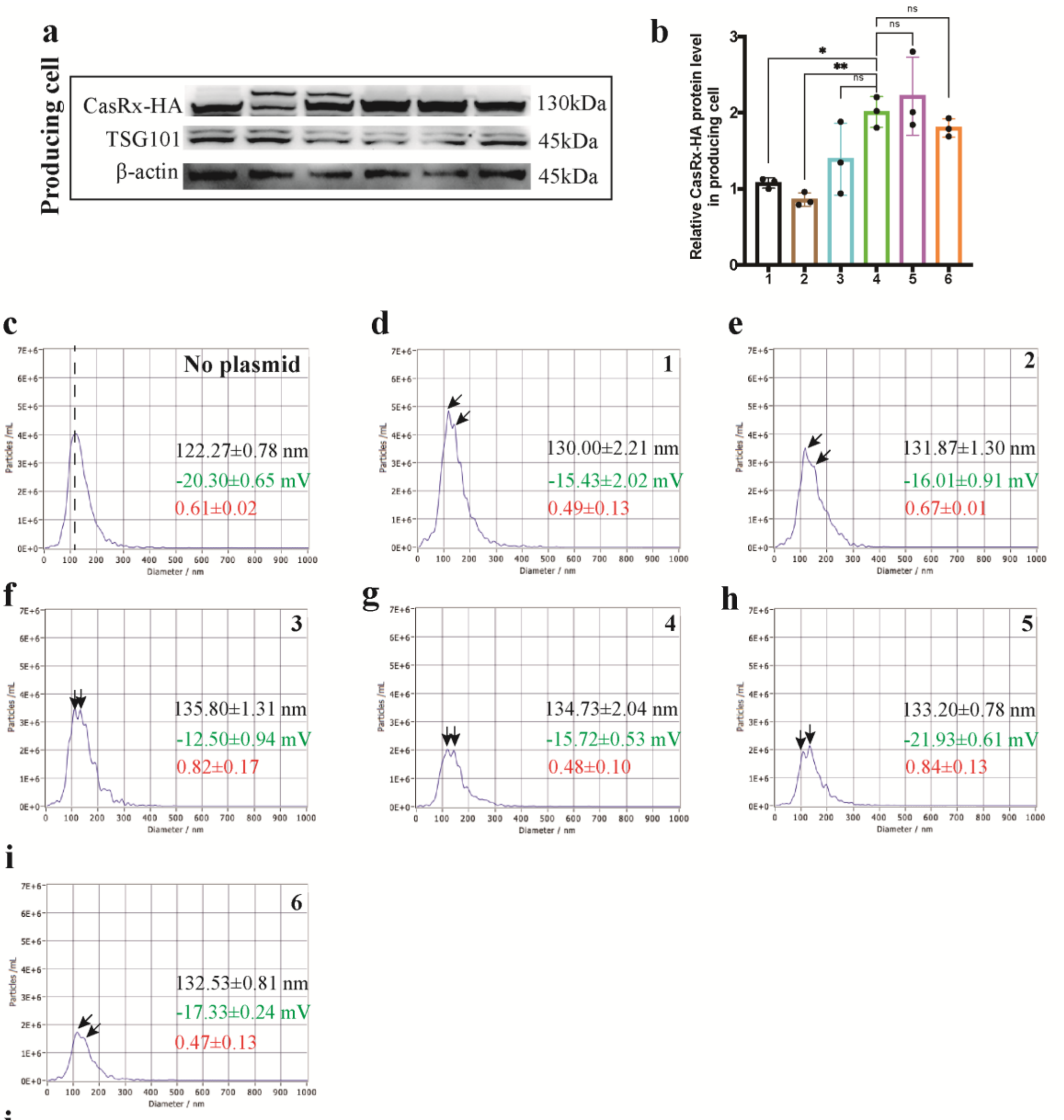

j

	No plasmid	1	2	3	4	5	6
**Mean particle size**	122.27±0.78 nm	130.00±2.21 nm	131.87±1.30 nm	135.80±1.31 nm	134.73±2.04 nm	133.20±0.78 nm	132.53±0.81 nm
**Zeta potential**	-20.30±0.65 mV	-15.43±2.02 mV	-16.01±0.91 mV	-12.50±0.94 mV	-15.72±0.53 mV	-21.93±0.61 mV	-17.33±0.24 mV
**Polydispersity index**	0.61±0.02	0.49±0.13	0.67±0.01	0.82±0.17	0.48±0.10	0.84±0.13	0.47±0.13

Supplemental Fig. 2 | Nanoparticle tracking analysis (NTA), zeta potential, and polydispersity index of EVs extracted from different groups. a, Immunoblotting analysis comparing CasRx-HA levels in producing cells from different groups. β-actin served as control. **b,** Immunoblotting analysis comparing CasRx-HA levels in producing cells from different groups. β-actin served as control. **c,** NTA analysis of EVs from HEK293T cells without transfection of plasmid. The dashed line indicates a single peak of particle diameter. The mean particle size, zeta potential, and polydispersity index were shown in black, green, and red text. **d-i**, NTA analysis of EVs from HEK293T cells with transfection of corresponding plasmids in Figure 1a. The arrows indicate two peaks of particle diameter. The respective mean particle size, zeta potential, and polydispersity index were shown in black, green, and red text. **j,**

Summary of mean particle size, zeta potential, and polydispersity index of seven different EVs. ****$p < 0.0001$, ***$p < 0.001$, **$p < 0.01$, *$p < 0.05$. The data are expressed as the mean ± SD.

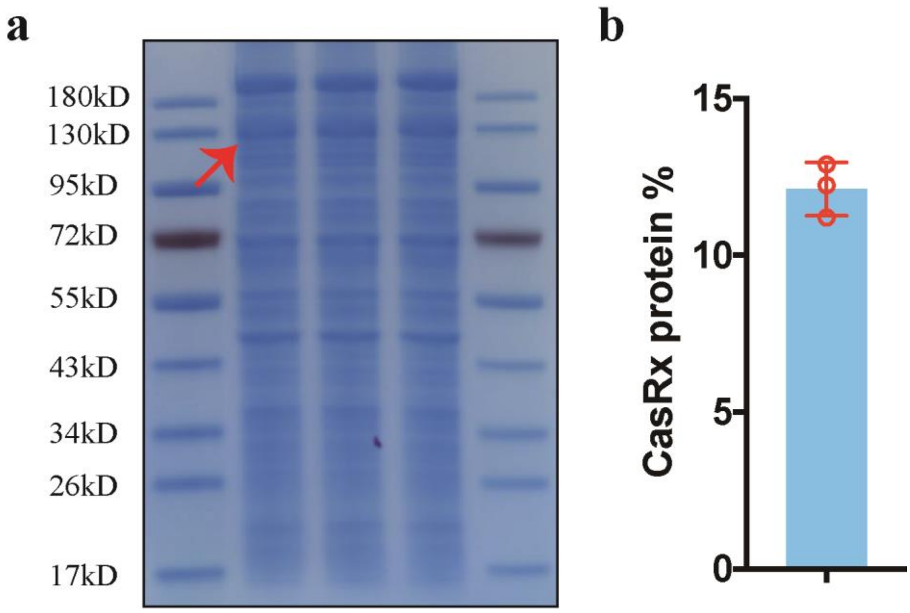

Supplemental Fig. 3 | The Coomassie brilliant blue staining of tPA-CasRx EVs. a, The Coomassie brilliant blue staining of three repeats of tPA-CasRx EVs. The arrow indicates CasRx band. **b,** Statistical results of proportion of CasRx in tPA-CasRx EVs. The data are expressed as the mean ± SD.

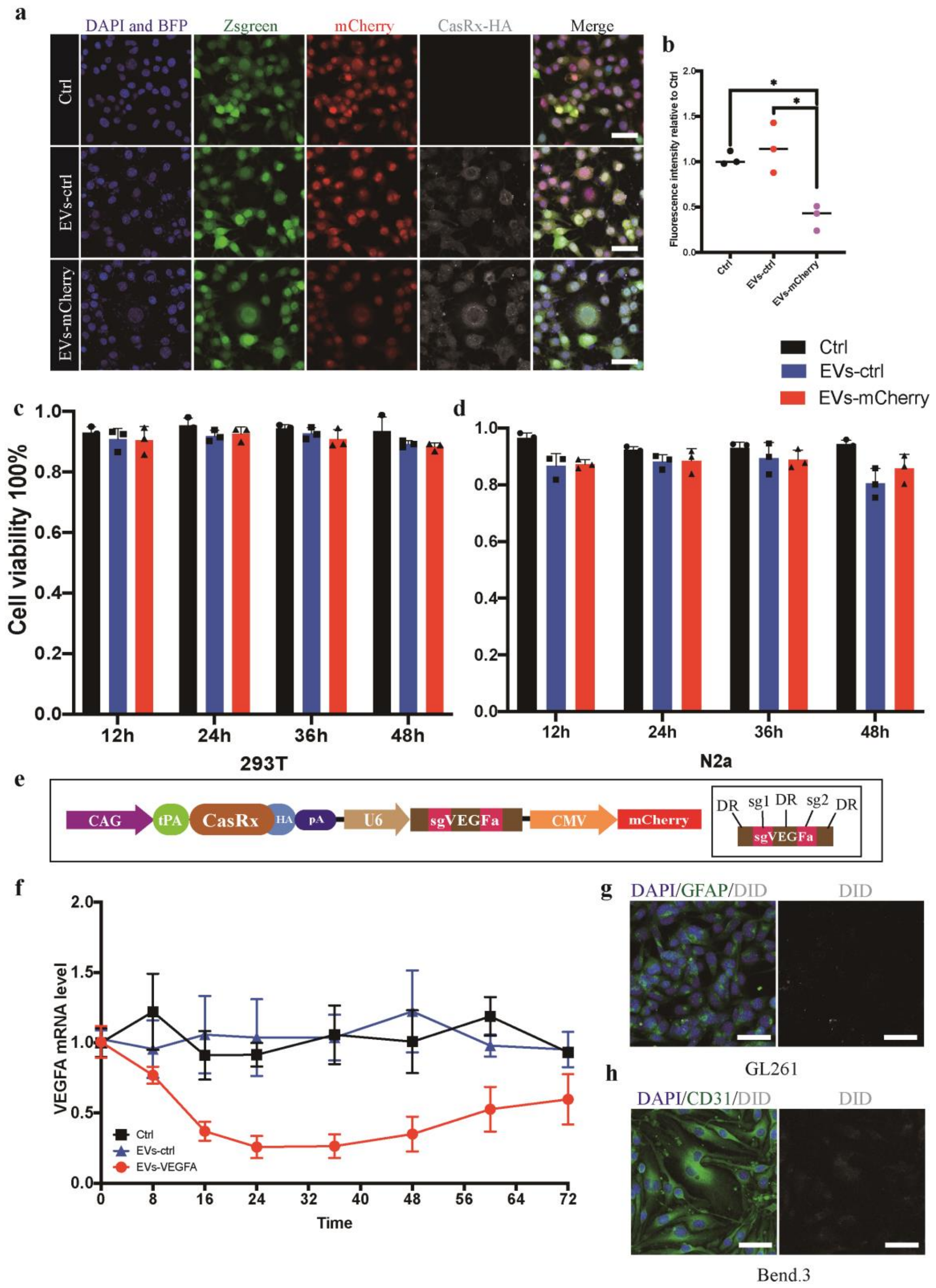

Supplemental Fig. 4 | The engineered EVs-CasRx/gRNA system suppresses exogenous and endogenous gene expression *in vitro*. **a,** Representative immunofluorescence images of Neuro-2a cells that received different treatments. Scale bar = 50 μm. **b,** Statistic result of mCherry MFI between three different groups. **c-d,** Cell viability of HEK293T and N2a cells receiving EVs treatment. **e,** Schematic illustration of the EVs plasmid design that targeting VEGFA. The detailed design of

gRNAs was shown in right box. **f,** The change of VEGFA mRNA levels of HEK293T over time demonstrated that the VEGFA mRNA levels decreased within 24 hours but gradually returned to the original level after 36 hours. **g-h,** Representative immunofluorescence images of GL261 and Bend.3 that received EVs treatment. ****p < 0.0001, ***p < 0.001, **p < 0.01, *p < 0.05. The data are expressed as the mean ± SD.

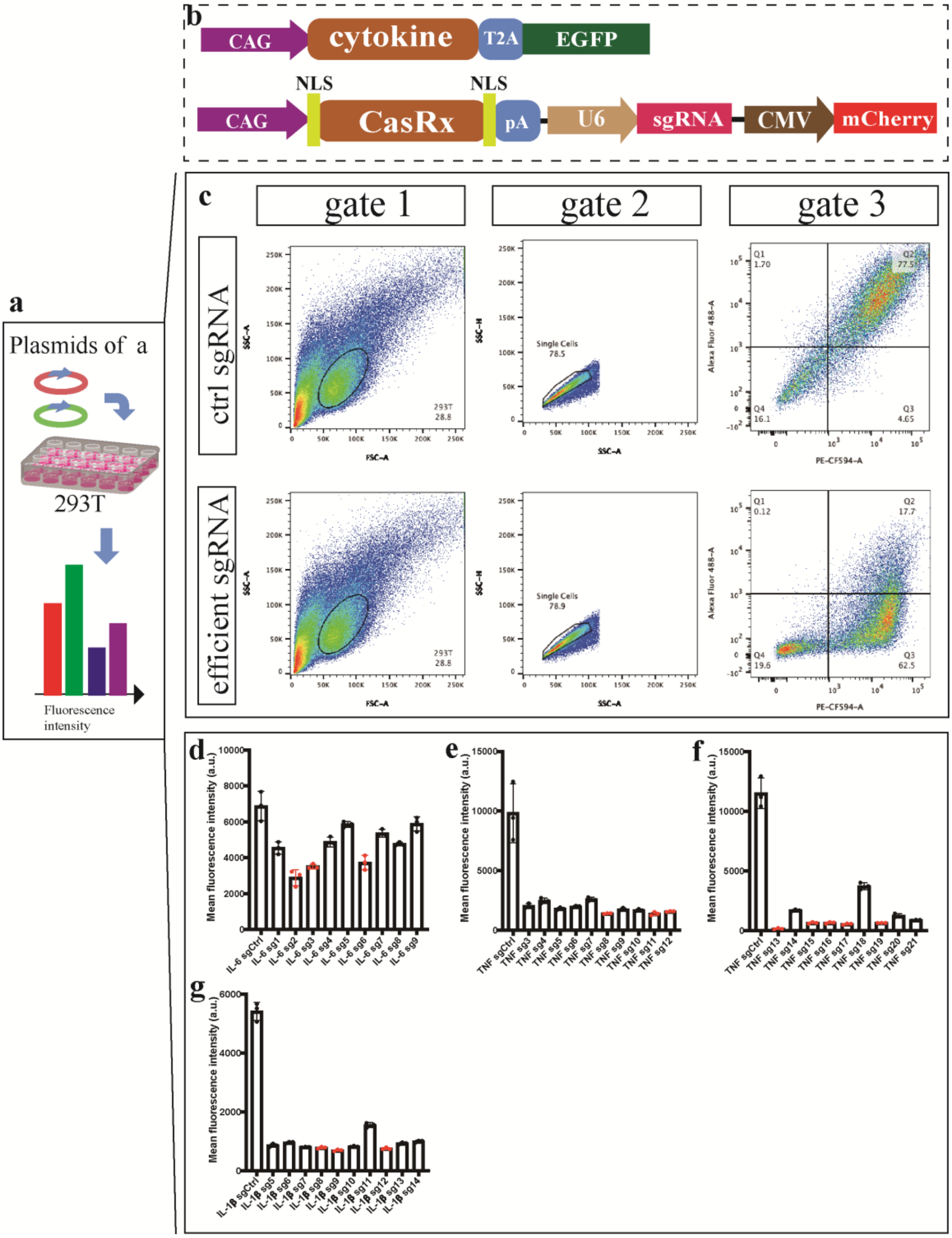

Supplemental Fig. 5 | Strategy 1 used to preliminarily screen for effective gRNAs targeting cytokines. a, The schematic image of experimental design. **b**, A schematic

of the two vectors preliminarily screen for effective gRNAs targeting cytokines. **c,** Representative images of the gating strategy and results for preliminary screening. **d,** Statistic results of MFI of EGFP co-expressed with IL-6 showing that sg2, sg3, and sg6 were potential effective gRNAs. **e-f,** Statistic results of MFI of EGFP co-expressed with TNF showing that sg8, sg11, sg12, sg13, sg15, sg16, sg17 and sg19 (rounds 1 and 2) were potential effective gRNAs. **g,** Statistic results of MFI of EGFP co-expressed with IL-1β showing that sg8, sg9, and sg12 were potential effective gRNAs. ****$p < 0.0001$, ***$p < 0.001$, **$p < 0.01$, *$p < 0.05$. The data are expressed as the mean ± SD.

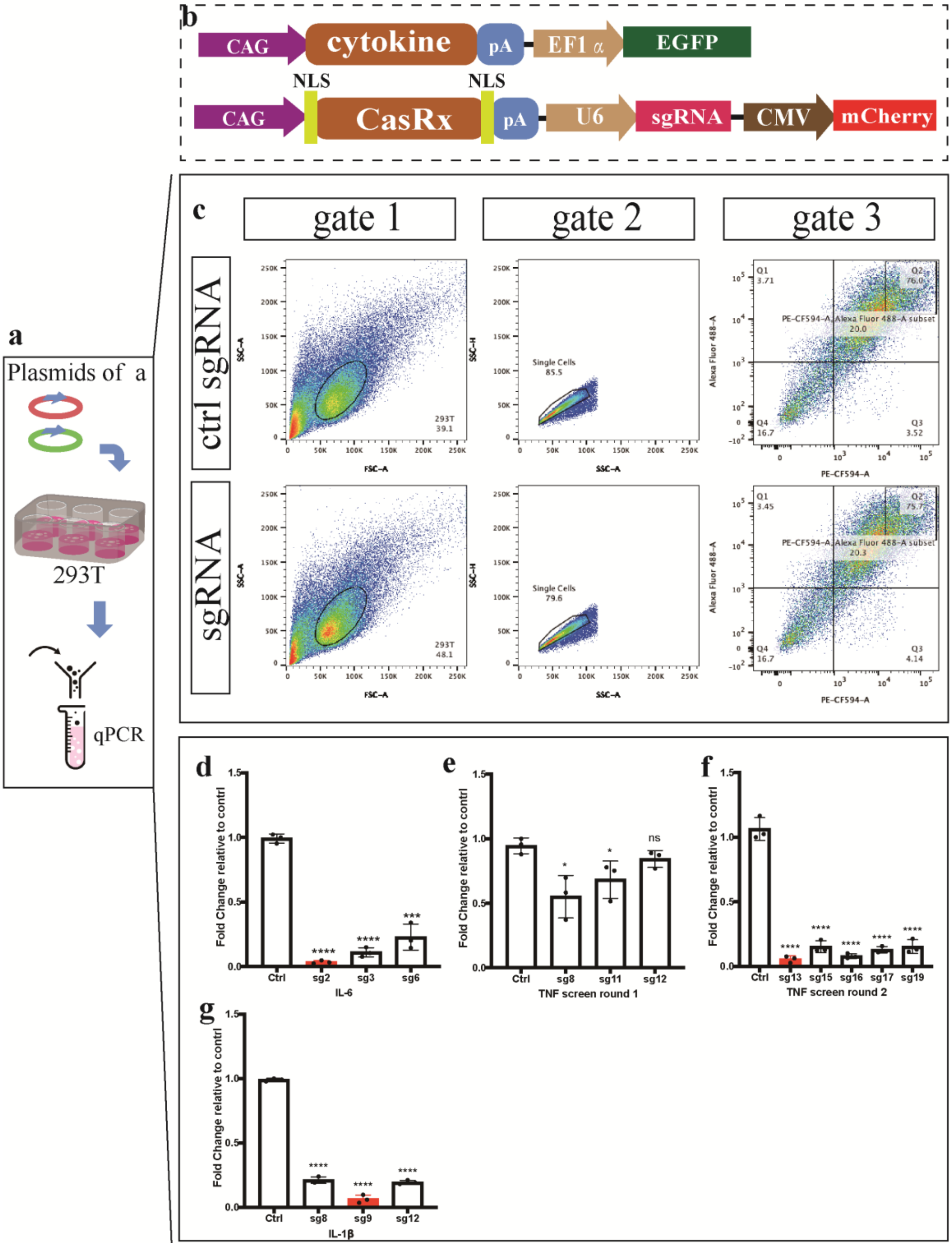

Supplemental Fig. 6 | Strategy 2 used to screen out effective gRNAs targeting cytokines. a, The schematic image of experimental design. **b,** A schematic of the two vectors to screen out most effective gRNAs targeting cytokines. **c,** Representative images of the gating strategy and results to screen out most effective gRNAs. **d**, Knockdown efficiency of different gRNAs for IL-6 showing that sg2 had the most potent knockdown efficiency. **e-f,** Knockdown efficiency of different gRNAs for TNF showing that sg13 had the most potent knockdown efficiency. **g,** Knockdown efficiency of different gRNAs for IL-1β showing that sg9 had the most potent knockdown efficiency. ****$p < 0.0001$, ***$p < 0.001$, **$p < 0.01$, *$p < 0.05$. The data are expressed as the mean ± SD.

a

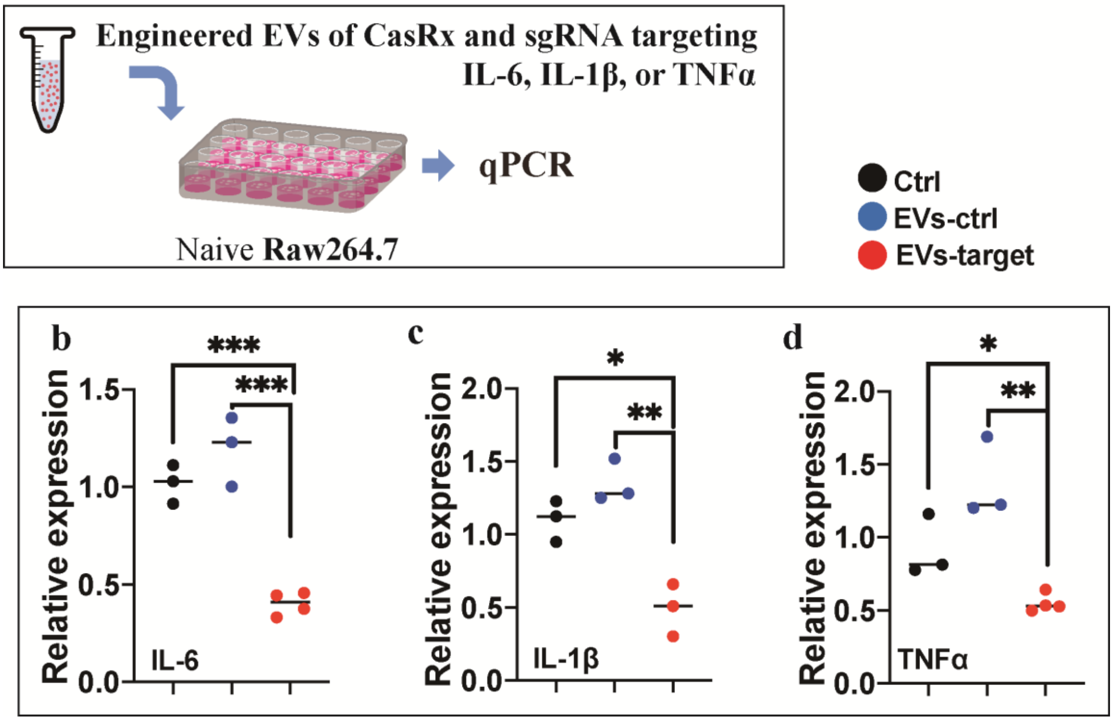

Supplemental Fig. 7 | CasRx/gRNA delivered by EVs could knock down cytokine expression in naïve Raw264.7 cells. a, The schematic image of experimental design. **b-d,** Statistic results of relative mRNA level of IL-6, IL-1β and TNF showed that EVs slightly upregulated the cytokine expression and CasRx/gRNA delivered by EVs could knock down IL-6, IL-1β and TNF efficiently. ****$p < 0.0001$, ***$p < 0.001$, **$p < 0.01$, *$p < 0.05$. The data are expressed as the mean ± SD.

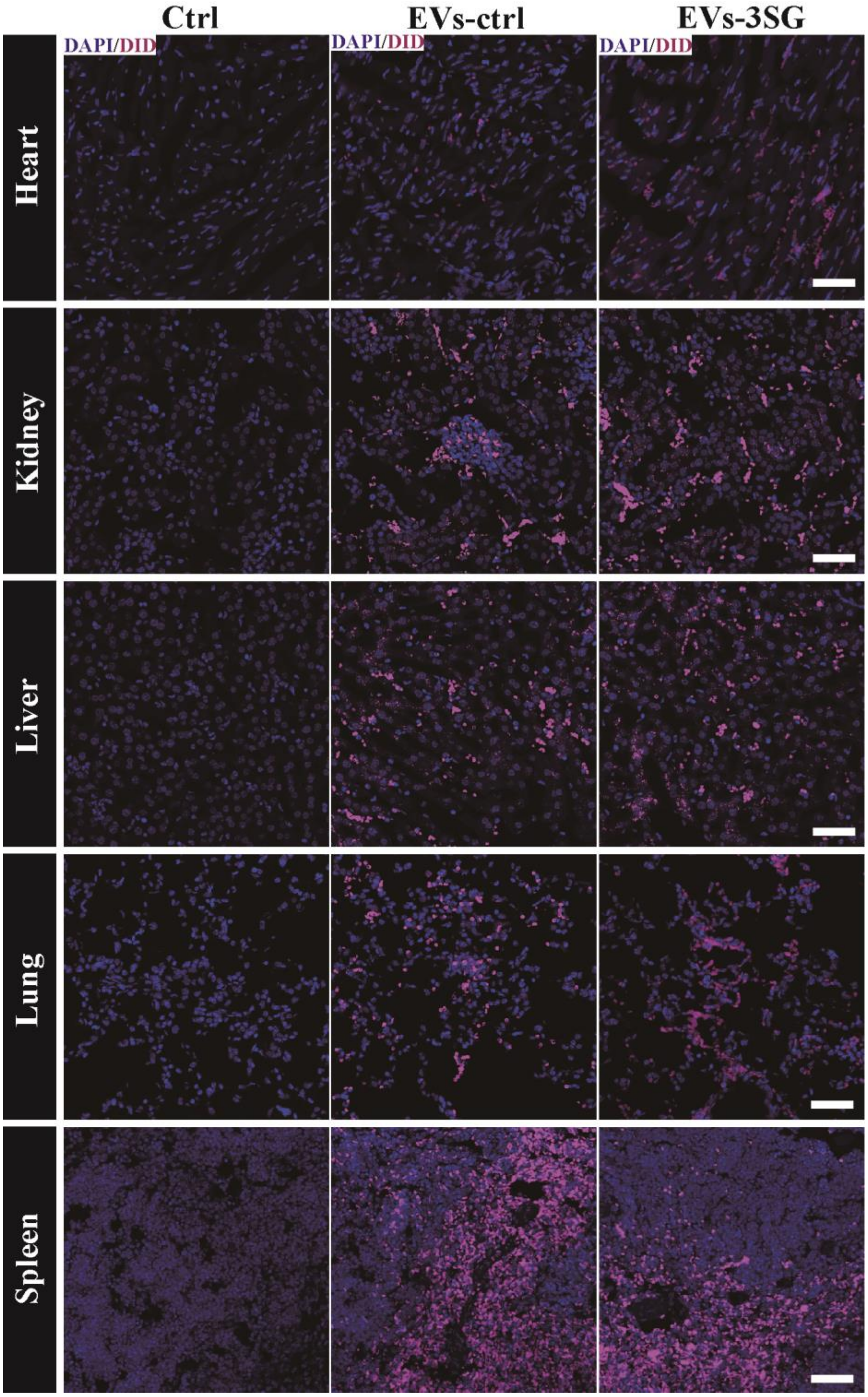

Supplemental Fig. 8 | Biodistribution of EVs. Representative immunofluorescence images of tissue from the heart, kidney, liver, lung and spleen. Scale bar = 100 μm.

Supplemental Table 1. The sequence of single-guide RNA used in this study.

**Single-guide RNA used in this study**	
**Primer**	**Sequence**
sg-ctrl	gggtcttcgatattcaagcgtcggaagacct
sg-mCherry	caagtgggagcgcgtgatgaacttcgagga
sg-VEGFA	ggtactcctggaagatgtccaccagggtctc-DR-gtgctgtaggaagctcatctctcctatgtg
**IL-1β screen**	**Sequence**
sg5	cctgatgagagcatccagcttcaaatctcg
sg6	cgcagcagcacatcaacaagagcttcagg
sg7	atcactcattgtggctgtggagaagctgtgg
sg8	ggaccttccaggatgaggacatgagcacctt
sg9	gatgatgataacctgctggtgtgtgacgtt
sg10	tagacaactgcactacaggctccgagatgaa
sg11	ctccacctcaatggacagaatatcaaccaa
sg12	ggacagaatatcaaccaacaagtgatatt
sg13	ctatacctgtcctgtgtaatgaaagacggca
sg14	ctatacctgtcctgtgtaatgaaagacggca
**IL-6 screen**	**Sequence**
sg1	gggactgatgctggtgacaaccacggcctt
sg2	cacagaggataccactcccaacagacctgt
sg3	cacaagtcggaggcttaattacacatgttct
sg4	ccagagatacaaagaaatgatggatgcta
sg5	gcctattgaaaatttcctctggtcttctggag
sg6	cctctggtcttctggagtaccatagctacc
sg7	catatcttcaaccaagagataagctggagt
sg8	caaccaagagataagctggagtcacagaaggag
sg9	ctaattcatatcttcaaccaagagataagct
**TNF screen**	**Sequence**

sg3	gagcacagaaagcatgatccgcgacgtggaa
sg4	tccagaactccaggcggtgcctatgtctcag
sg5	ccaggcggtgcctatgtctcagcctcttct
sg6	gcctatgtctcagcctcttctcattcctg
sg7	gagaagttcccaaatggcctccctctcat
sg8	ctcatcagttctatggcccagaccctcaca
sg9	ggaggagcagctggagtggctgagccagcg
sg10	cctggccaacggcatggatctcaaagacaa
sg11	agtggtgccagccgatgggttgtaccttgt
sg12	gctatctcataccaggagaaagtcaacctc
sg13	gacaagcctgtagcccacgtcgtagcaaa
sg14	ctcattcctgcttgtggcaggggccaccacg
sg15	ctgtctactgaacttcggggtgatcggt
sg16	cggggtgatcggtccccaaagggatgagaag
sg17	caagggacaaggctgccccgactacgtgct
sg18	ctcttcaagggacaaggctgccccgacta
sg19	gtctactcccaggttctcttcaagggac
sg20	gtaccttgtctactcccaggttctctt
sg21	gcggagtccgggcaggtctactttggagt

Supplemental Table 2. The qPCR primer used in this study

Primers	Sequence
IL-6 Forward	CAACGATGATGCACTTGCAGA
IL-6 Reverse	TCTGTGACTCCAGCTTATCTCTTG
TNF Forward	AGGCACTCCCCCAAAAGATG
TNF Reverse	CCACTTGGTGGTTTGTGAGTG
IL-1β Forward	AATGCCACCTTTTGACAGTGAT
IL-1β Reverse	CCATGAGTCACAGAGGATGGG
VEGFA-mouse Forward	ACATCTTCAAGCCGTCCTGT
VEGFA-mouse Reverse	TTGACCCTTTCCCTTTCCTCG
VEGFA-human Forward	CCATCCAATCGAGACCCTGG
VEGFA-human Reverse	CACCAACGTACACGCTCCA
